# Supplementary material for: Intersectional tension: a qualitative study of the effects of the COVID-19 response on survivors of violence against women in urban India
Source: BMJ Open. 2021 Sep 27;11(9):e050381. doi: 10.1136/bmjopen-2021-050381 (PMC8478579; doi:10.1136/bmjopen-2021-050381)
Supplement: Supplementary data [file bmjopen-2021-050381supp001.pdf]

SNEHA Program on Prevention of Violence Against Women and Children

Counsellor telephone interviews with registered women clients

March 2020

Topic guide

We are interested in understanding how the Covid-19 situation has affected our clients.

During your phone conversation with the client, please ask her the series of questions below. Record or note what she says. Before you do this, take her consent for the recording (see below).

We are doing this for the NIHR Global Health Research Group on a package of care for the mental health of survivors of violence in South Asia.

We have approval for qualitative data collection from the University College London Research Ethics Committee (2744/007) and the Sangath Institutional Review Board (AN_2018_46).

Please remind the client that when she first consulted SNEHA she agreed that we could use information she provided for research purposes. We will make sure that anything she says is not identifiable and we will never include her name or other details in any information we share. We will use the information collected to improve the programme in times of pandemics like Covid-19 in the future. You can assure the client that her information will be kept anonymous and confidential and only used by the program.

Tell the client that she does not have to answer any question if she doesn't want to, and that whether or not she does will have no bearing on the way SNEHA supports her.

Ask her if she agrees to either:

Her answers to the questions being recorded on the phone.

Or:

You taking notes of the answers to the questions.

The questions

You may like to ask these questions early in the conversation as they are more general and less sensitive than the issues for which she has consulted and which you will be discussing during the call.

You can ask the questions later if the client wants to discuss a specific issue about her case. If she would prefer it, you can ask her for an exclusive appointment later to ask the interview questions.

You don't have to ask them in order or exactly as they are written: listen to what the client says and then probe until you understand.

How are you doing in the present situation?

Are you facing any kind of problems?

Has the Covid-19 crisis made your situation different from earlier times in your day-to-day life?

How is it with everyone staying at home?

Have your responsibilities changed compared to before the situation?

How are you managing things like food and money?

How is your family reacting to the situation?

Do you think the situation has affected you being able to talk to someone about your issues?

Has the crisis brought your family closer or has it created more problems?

Do you think counselling could be done better if you went to the counselling centre instead of talking like this on the phone?

Note down any particular observations or difficulties you have in the conversation.
