# Supplementary material for: Intersectional tension: a qualitative study of the effects of the COVID-19 response on survivors of violence against women in urban India
Source: BMJ Open. 2021 Sep 27;11(9):e050381. doi: 10.1136/bmjopen-2021-050381 (PMC8478579; doi:10.1136/bmjopen-2021-050381)
Supplement: Author's manuscript [file bmjopen-2021-050381.draft_revisions.pdf]

BMJ Open

BMJ Open is committed to open peer review. As part of this commitment we make the peer review history of every article we publish publicly available.

When an article is published we post the peer reviewers' comments and the authors' responses online. We also post the versions of the paper that were used during peer review. These are the versions that the peer review comments apply to.

The versions of the paper that follow are the versions that were submitted during the peer review process. They are not the versions of record or the final published versions. They should not be cited or distributed as the published version of this manuscript.

BMJ Open is an open access journal and the full, final, typeset and author-corrected version of record of the manuscript is available on our site with no access controls, subscription charges or pay-per-view fees (http://bmjopen.bmj.com).

If you have any questions on BMJ Open's open peer review process please email info.bmjopen@bmj.com

BMJ Open

A hierarchy of stress: a qualitative study of the effects of the Covid-19 response on survivors of violence against women in urban India

Journal:	*BMJ Open*
Manuscript ID	bmjopen-2021-050381
Article Type:	Original research
Date Submitted by the Author:	19-Feb-2021
Complete List of Authors:	Huq, Mita; UCL, Institute for Global Health<br>Das, Tanushree; Society for Nutrition, Education & Health Action, Program on Prevention of Violence Against Women and Children<br>Devakumar, Delan; University College London, Institute for Global Health<br>Daruwalla, Nayreen; Society for Nutrition, Education & Health Action, Program on Prevention of Violence Against Women and Children<br>Osrin, David; University College London, Institute for Global Health
Keywords:	COVID-19, Public health < INFECTIOUS DISEASES, MENTAL HEALTH, QUALITATIVE RESEARCH

SCHOLARONE™
Manuscripts

BMJ

I, the Submitting Author has the right to grant and does grant on behalf of all authors of the Work (as defined in the below author licence), an exclusive licence and/or a non-exclusive licence for contributions from authors who are: i) UK Crown employees; ii) where BMJ has agreed a CC-BY licence shall apply, and/or iii) in accordance with the terms applicable for US Federal Government officers or employees acting as part of their official duties; on a worldwide, perpetual, irrevocable, royalty-free basis to BMJ Publishing Group Ltd ("BMJ") its licensees and where the relevant Journal is co-owned by BMJ to the co-owners of the Journal, to publish the Work in this journal and any other BMJ products and to exploit all rights, as set out in our licence.

The Submitting Author accepts and understands that any supply made under these terms is made by BMJ to the Submitting Author unless you are acting as an employee on behalf of your employer or a postgraduate student of an affiliated institution which is paying any applicable article publishing charge ("APC") for Open Access articles. Where the Submitting Author wishes to make the Work available on an Open Access basis (and intends to pay the relevant APC), the terms of reuse of such Open Access shall be governed by a Creative Commons licence – details of these licences and which Creative Commons licence will apply to this Work are set out in our licence referred to above.

Other than as permitted in any relevant BMJ Author's Self Archiving Policies, I confirm this Work has not been accepted for publication elsewhere, is not being considered for publication elsewhere and does not duplicate material already published. I confirm all authors consent to publication of this Work and authorise the granting of this licence.

For peer review only

A hierarchy of stress: a qualitative study of the effects of the Covid-19 response on survivors of violence against women in urban India

AUTHORS

Mita Huq

Institute for Global Health, University College London, London, WC1N IEH, UK

mita.huq.19@ucl.ac.uk

Tanushree Das

Program on Prevention of Violence Against Women and Children, SNEHA, Mumbai, Maharashtra, 400017, India

tanushree.das@snehamumbai.org

Delan Devakumar

Institute for Global Health, University College London, London, WC1N IEH, UK

d.devakumar@ucl.ac.uk

Nayreen Daruwalla

Program on Prevention of Violence Against Women and Children, SNEHA, Mumbai, Maharashtra, 400017, India

nayreen@snehamumbai.org

David Osrin

Institute for Global Health, University College London, London, WC1N IEH, UK

d.osrin@ucl.ac.uk

WORD COUNT 4996

For peer review only

ABSTRACT

Objectives

There is concern worldwide that the physical and emotional constraints that arise from efforts to address the SARS-CoV-2 pandemic have affected the frequency and intensity of domestic violence against women. Residents of urban informal settlements faced particularly stringent conditions during the response in India. We spoke with survivors of domestic violence in urban informal settlements in Mumbai, with two objectives: to understand how the pandemic and subsequent lockdown changed their needs and affected their vulnerabilities, and to recommend programmatic responses.

Design

Qualitative interviews and framework analysis.

Setting

A non-government support program for survivors of violence against women, providing services mainly for residents of informal settlements.

Participants

During telephone conversations with registered women survivors of violence, counsellors asked about the effects of Covid-19 on their daily and family life, their ability to speak with someone, and their counselling preferences.

Results

For 720 clients interviewed between 1st April and 30th July 2020, the major concerns centred on financial stress, interrupted livelihoods, and food insecurity. Many had experienced stress from changes in household composition and increased responsibilities that fell primarily on women. The effects on domestic violence depended on current household structure, but were not limited to intimate partners. Clients supported the use of telephonic counselling, although mostly as an interim rather than longer-term solution.

Conclusions

Our findings underline the importance of financial precarity and access to resources and support. The pandemic has increased existing stress around poverty and gendered reproductive labour. Responses must focus on structural vulnerability, distributing resource-based support, prioritising the continued availability of services for survivors of violence, and expanding access to social networks. Decision-makers must be aware of the gendered and intersectional effects of interventions and must include residents of informal settlements who are survivors of domestic violence in the planning and implementation of public health strategies.

KEYWORDS

Covid-19; Domestic Violence; Gender-based violence; poverty areas; Mumbai; India

For peer review only

For peer review only - http://bmjopen.bmj.com/site/about/guidelines.xhtml

Article summary

Strengths and limitations of this study

- Amid concerns that the response to the SARS-CoV-2 pandemic has increased the incidence and severity of domestic violence against women, counsellors in touch with survivors were able to talk to over 700 women about their experiences.
- All the participants were residents of urban informal settlements in which stringent measures to limit the spread of Covid-19 were implemented.
- Survivors' concerns centred on financial precarity, food insecurity, and increased gendered responsibilities to provide for household members.
- All the participants had already sought help from services and we were therefore unable to assess the incidence of new cases of violence against women.

For peer review only

For peer review only - http://bmjopen.bmj.com/site/about/guidelines.xhtml

BACKGROUND

As governments implemented social and systems measures to address the spread of SARS-CoV-2, organisations working to prevent violence against women became concerned that the measures might themselves give rise to or escalate violence, a situation described as a syndemic,[1] danger in danger,[2] a pandemic within a pandemic,[3] a pandemic paradox,[4] or a shadow pandemic.[5] The concern is informed by three strands of evidence. The first is that public health emergencies have been shown repeatedly to be followed by increases in violence against women and girls.[6 7] The second is that states and civil society organisations noted large increases in calls to domestic violence helplines,[8 9 10] and apparent increases in frequency and severity,[7 11-13] at the same time as the numbers of face-to-face consultations fell.[14] The third concern was that the conditions imposed by the pandemic were intuitively likely to increase household and interpersonal conflict. As early as March 2020, the World Health Organization (WHO) flagged the concern that measures such as quarantine and lockdown could increase the risk of violence against women,[15] a concern supported by a rapid assessment by UN Women.[8]

The measures implemented in India in response to the Covid-19 pandemic were sudden and stringent, particularly in urban informal settlements (slums). Residents of such settlements, in which ~40% of Mumbai's households are located, were seen as especially vulnerable because of density and overcrowding, insubstantial housing, and insufficient water and sanitation.[16 17] An average of five people share one or two rooms, most rely on shared toilets, and around two-thirds need to leave the house to get water. The government of Maharashtra announced a lockdown in Mumbai on 23rd March 2020, for an initial 21 days with subsequent extensions. Major informal settlements were quarantined as red zones,[18] their access barricaded, public toilets disinfected, and a police-enforced curfew instituted. Residents were confined to their homes unless they faced medical emergencies, transport services and vehicular movement curtailed, and industry and retail outlets closed. Residents were screened for SARS-CoV-2 through door-to-door visits by public and private sector healthcare workers, with an emphasis on test and trace. Quarantine centres were set up in schools and halls, with free food and healthcare.[17] Fear when individuals and families were put into this institutional quarantine was common and accompanied by a degree of community stigma.

These measures were commended for preventing a surge in cases in areas where the potential spread of SARS-CoV-2 was particularly concerning.[17 19] An increase in violence against women was, however, well-recognised in the media and a small number of studies, but the breadth of literature exploring how and why survivors experience domestic violence during the Covid-19 pandemic has so far been limited.[20] This has been at least partly the result of reduced capacities and interrupted monitoring and evaluation activities on the part of organisations working to prevent violence.[8 15 21]

We interviewed survivors of domestic violence in urban informal settlements in Mumbai, with two objectives: to understand how the Covid-19 pandemic and subsequent lockdown changed their needs and affected their vulnerabilities, and to recommend programmatic responses to domestic violence in light of a pandemic and the lockdown strategies used to mitigate it.

METHODS

Setting

Based at the non-government organization SNEHA (Society for Nutrition, Education and Health Action), the Program on Prevention of Violence Against Women and Children has provided support to over 13 000 survivors of violence against women since 2000. Services are open to all, but particularly residents of Mumbai's informal settlements, and are delivered from five community and three public hospital counselling centres. We support survivors of violence through a combination of in-house services and liaison. In-house human resources

For peer review only

For peer review only - http://bmjopen.bmj.com/site/about/guidelines.xhtml

include community outreach workers, counsellors, legal advisors and lawyers, and psychologists. These are complemented by liaison with hospital practitioners, the police, District Legal Aid services, shelters, and psychiatrists.

Participants

Interviewees were women clients already registered with our domestic violence services who had been interacting with counsellors prior to the lockdown. As the response to the pandemic unfolded, we moved our counselling and crisis intervention services online through existing crisis helplines and an email facility. All our counselling centres were provided with helplines which women and children survivors could call for assistance. Community volunteers gave out the numbers to women who were suffering violence. At the same time, counsellors contacted their existing clients and offered to provide follow-up telephonically. We developed a protocol for this follow-up, based on existing safety protocols and emphasising confidentiality and the same client-centred approach followed in face-to-face sessions.

Data collection

Eleven female counsellors with masters-level education in social work or psychology and between two and ten years of experience of supporting clients from our centres conducted interviews after receiving project-specific training. Clients had registered with them through self-report, referral from agencies such as the police, clinicians, or non-government organizations, or referral by SNEHA community organisers or volunteers (*sanginis*) trained to identify and engage with survivors.[22] Participants were contacted by their counsellors for telephone follow-up between March and May 2020. Counsellors explained that, as part of their conversations, they were doing a study to understand women's experiences during the lockdown. With agreement from clients, they administered a semi-structured interview about how Covid-19 had affected their daily life, family life, ability to speak with someone, and their counselling preferences. Interviews were conducted during working hours. If a client did not answer the phone, nearby field staff, volunteers, or women's group members would visit her and encourage her to contact her counsellor to indicate her availability. Counsellors provided all clients with information on Covid-19. If a family member answered the phone (or it seemed likely to the counsellor that someone was listening in), they said that the call was part of the Covid-19 prevention program and that they were interested in the family's emotional health. Key questions were provided to counsellors, who recorded responses in field notes. Interviews were conducted in Hindi and Marathi and subsequently summarised in English by bilingual team leaders. Interviews were part of counselling calls that lasted up to an hour.

All potential identifiers were removed from the dataset.

Data analysis

We used the framework approach.[23 24] The familiarisation process began with reading of all records and elimination of duplicates. The data were explored and indexed in NVivo 12.06.0. Initial codes and themes were derived deductively from the interview questions and familiarisation process. The indexing process expanded this framework by inductively adding themes, sub-themes, and categories. Two researchers approached the data independently (MH and DO). The final iteration of indexing and data exploration assessed the thematic framework for distinction between codes, themes, and categories. The outputs of this process were charted in a framework matrix for further analysis and interpretation.

Ethical considerations

The project was conducted within the NIHR Global Health Research Group on *A package of care for the mental health of survivors of violence in South Asia*, with approval for qualitative data collection from the University College London Research Ethics Committee (2744/007) and the Sangath Institutional Review Board (AN_2018_46). Clients gave signed consent for

For peer review only

anonymised or pseudonymised use of their data at initial registration. They were assured of confidentiality, particularly that information would not be shared with their family, community, or the media. Counsellors informed clients of their right to access their records, including for use in building legal evidence.

Patient and public involvement

Clients of our counselling centres for survivors of violence against women were first involved during telephonic discussions with counsellors. The research questions were a direct response to concerns about their safety and wellbeing during the response to the Covid-19 pandemic, and sought to understand their experiences and preferences for services. Dissemination will involve personal feedback of the findings to clients during subsequent counselling sessions and feedback during regular community mobilisation meetings in informal settlements across the city.

RESULTS

Between 1st April and 30th July 2020, counsellors attempted to contact 924 clients. They achieved substantive conversations with 720 clients (78%), on whose responses the analysis was based. We discuss their concerns, their experience of violence, and their preferences for access to services. Respondents' main worries were around livelihoods and financial stress, food insecurity, and changes in household composition and gendered responsibilities. Across the board, these changes had two kinds of effect: physical and cognitive.

Finances and food

Dominant in women's narratives were problems in meeting basic needs for money and food. Families living in informal settlements depend largely on a mosaic of livelihoods in the informal sector and most women said that the lockdown had caused work to be suspended. Restrictions on movement meant that daily earners could not go to work: "I used to do domestic work but am now unable to go to work. My employers have been calling me to say that because I'm from **** and Covid-19 cases are high here, they don't want me to join work for another 6 months. I'm worried about finances" (ID 840, 20 April 2020). A few days of lockdown led many families to draw on their limited savings. "We're running out of savings. My husband's boss is not able to pay him right now but is providing him with the basics so he's staying at the job. But my kids and I are struggling to make ends meet" (ID 735, 13 April 2020).

The only safety net was the government ration system, itself compromised by halts in supply and the fencing off of communities. Families who were eligible for rations became more dependent on them, but the quantity and quality of rations and food in the shops was compromised by supply problems. "The food resources have been insufficient, as well as the whole family is present and two meals for all become a little tough with this current crisis" (ID 791, 22 April 2020). Food insecurity therefore resulted from both lack of money and unavailability of food: "All shops are closed. The area is sealed. We are managing with the rations we get from the ration shop. Other shops are closed. Around 15 days back a lady had come to ask for our contact numbers to provide help, but we haven't heard from her or anyone so far" (ID 818, 9 April 2020). Coupled with inconsistent or insufficient government and non-government assistance, these pressures made members of food insecure families leave the home in contravention of lockdown policy to seek food, exposing themselves to SARS-CoV-2 and punitive action by law enforcement. Cognitively, food insecurity and poverty were potent stressors and their effects were magnified by the limits set to mobility, which led to feelings of helplessness and lack of opportunity to decompress. "My husband is getting violent and I'm alone at home and neither of us can leave to cool down. I feel trapped" (ID 14, 4 August 2020).

Household composition and responsibilities

For peer review only - http://bmjopen.bmj.com/site/about/guidelines.xhtml

The effects of changes in household structure and function developed on the background of insecurity in terms of basic needs. They varied markedly, but three general situations were described: women were living separately from their husbands and marital families, they were living with their husbands in nuclear or marital families, or they were living alone or with their children. Some of these arrangements were already in place before the lockdown. Survivors of violence had often separated from the perpetrators and the experience was not new. What respondents said they found stressful were changes in household arrangements: unaccustomed cohabitation with their natal family, the need to spend the whole day with their partner, or a sudden influx of people into existing households. Again, these changes had both physical and cognitive effects. The most trying seemed to be the increase in household responsibilities that fell on women. Some of these related to hygiene: cleaning, disinfection, and childcare in an environment in which viral transmission was a major concern. “Household work increased. Now I am very conscious about hand washing and taking care of children” (ID 549 16 April 2020). The extra housework occasioned by more people in the household and children being off school was usually shouldered by women. “I have extra workload and no help, while if I expect help, my husband and mother-in-law abuse me” (ID 795, 23 April 2020). Women tended to be tasked with cooking and cleaning and men were tasked with leaving the house to get supplies. The cognitive effects of these responsibilities centred on weariness and frustration with gender norms, when families were “… using the lockdown for harassment and intimidation” (ID 29, 19 April 2020).

Respondents who also worked from home reported difficulty balancing employment with their caring responsibilities, saying that family members had heightened expectations that they would complete chores. “I have to work more than before and my daughter-in-law keeps saying that if I don’t work I am just another one using their resources” (ID 813, 19 May 2020). Reproductive labour ranked higher than productive labour and expectations could be triggers for abuse: “My natal family does emotional violence on me because I work. Because I am spending all day at home, my family expects me to do domestic work” (ID 287, 20 May 2020). Cognitively, some respondents who had moved in with their natal family for quarantine said that they felt guilty for burdening them as they were unable to contribute financially, and that their presence added to precarity. “I am staying with my natal family and, now that I am unable to contribute, I feel awkward and a burden to them. They never verbalized it, but the non-verbal gestures are enough to understand” (ID 800, 04 May 2020).

Domestic violence

22% of respondents had suffered violence since the imposition of lockdown: “My husband has become better than before since we moved away. But he still does hit me” (ID 762, 24 April 2020). The effect of spending time together could be positive or negative, depending on household structure and interpersonal relationships. For some women the lockdown had been positive. Families enjoyed spending more time together than usual: “I love taking care of my family and being with them. Glad they support me so much. We're very happy we're getting quality time together (ID 725, 4 July 2020). Previously acrimonious relations sometimes improved: “… My husband came closer to me and my daughter, taking care of both of us” (ID 69, 3 April 2020).

It was possible for violence to decrease as a result of wariness rather than benevolence, when the abuser relied on the respondent: “My husband fights with me all the time. He constantly bickers. He's not beating me right now because he knows he can't do without me for now” (ID 710, 3 April 2020). Others experienced heightened tension due to a combination of overcrowding in small spaces, precarity, and stress. “… tension and stress due to lack of money and resources. Sometimes we fight with each other, but … I’m not able to go outside…” (ID 61, 3 April 2020). The negative effects were often driven by hardship and mitigated by wherewithal. “Barely being able to manage. My husband came and fought with me a lot a couple of days ago. After that now the shop below my house is giving me stuff on loan” (ID 709, 3 April 2020). The impact of lockdown on domestic violence was notable when survivors were unable to leave their home to escape abuse, for several reasons: more

For peer review only

exposure to the abuser(s), higher stress levels ("tension"), and heightened expectations. Although all the respondents had experienced domestic violence, some experienced physical abuse for the first time.

Respondents also reported an increase in frequency and severity of domestic violence, and the use of lockdown to control them. "It has given my husband more power. It's very difficult for us" (ID 715, 4 April 2020). Money was withheld or taken from them and abusers controlled their movements indoors, forced them out of the house, or isolated them socially. "Since March, I'm not allowed to enter the kitchen. They bring me food three times a day to my room" (ID 854, 11 May 2020). A few survivors said that their partners had not allowed them to access medical care on the pretext of acquiring infection. Other respondents received threats of violence from their abusers which prevented them from expressing themselves to anyone. Counsellors sometimes noted reticence in women's answers to questions: "Client's husband was there and so she was being a little calculating about what she was saying" (counsellor note, ID 750, 17 April 2020). There was also concern for children's wellbeing because fights, harassment, or physical violence that would otherwise have occurred while children were at school or outside the home now happened in front of them. "Initially he used to drink at night after work and hit me, when the kids were sleeping or not at home, but now they witness it which makes it worse for me all together" (ID 789, 15 April 2020).

The mental health concerns of respondents during lockdown centred on stress arising from income loss. Most were accustomed to struggling to meet their family's basic needs and domestic violence was one of a broad range of unacceptable burdens: "… Our problems are so much that none of this seems to be a big deal" (ID 739, 13 April 2020). Stress from experience of hardships was escalated by small living spaces, overcrowded homes, lack of privacy, or isolation. Lack of privacy and time alone exacerbated tension in large families, whereas loneliness was reported by women who lived alone or were being forcibly isolated or neglected. This was particularly concerning for respondents in abusive situations in need of counselling services, where the abuser was always around or monitoring their calls, or individuals whose emotional support came from outside the home such as neighbours, friends, or family members. "Before this situation, I spent some time with my neighbours to share my feelings and thoughts, but now due to more housework, I'm not able to manage more time for myself" (ID 62, 3 April 2020). Respondents described hesitancy to disclose their emotions to others for fear of being judged or misunderstood, or limited access to a phone. This hesitancy was shaped by the pandemic in that respondents felt that it was their responsibility to handle their mental health since the pandemic had already brought enough stress and fear to those around them. "I don't share with anyone. I don't want to trouble them. I don't talk to my family and I don't have any friends here. So I keep everything to me" (ID 729, 4 August 2020).

Lockdown changed the availability of alcohol, leading to improvements in some households, or a rise in tension in others due to withdrawal symptoms such as irritability. "It has created more issues and made it worse as my husband has been drinking the whole day and speaks only using abusive words and he had also abused my kids" (ID 789, 15 April 2020). For those reporting improvement, it was primarily through less drinking and more time spent caring for the family. "My husband has stopped drinking so there is some peace at home" (ID 828, 16 April 2020). In some households where drinking persisted, not only were tensions heightened at home, but abusers would leave the house to find alcohol. This caused further worries about exposure to SARS-CoV-2 and added to household fears for health and wellness.

Preferences for support

All of the respondents were survivors of violence with prior connections to counsellors. During the interviews, they recognised the importance of distanced counselling services during the pandemic, and generally found telephone discussion a good option. Some

For peer review only - http://bmjopen.bmj.com/site/about/guidelines.xhtml

actually preferred it to in-person meetings, saying that it overcame geographic and physical barriers and time constraints that had prevented them from visiting the centre. Nevertheless, most said that they would prefer to return to the counselling centre when it was safe. Respondents who reported substantial changes to their daily life said that they were unable to talk at home because of lack of privacy, family members listening in, higher risk of violence if discovered, or limited access to a phone. They said that in-person counselling was better because they felt more comfortable and articulate at the counselling centre and the services would be better. Respondents who did not report increased violence agreed with this, but needed different types of services from the centre, such as psychological counselling for family members other than themselves or legal guidance. Legal guidance was an especially common concern due to the halt in proceedings and inability to complete divorce or other case paperwork.

DISCUSSION

During the response to the Covid-19 pandemic, survivors of domestic violence in Mumbai's urban informal settlements faced - as well as fears about the pandemic itself - heightened financial and food insecurity, gendered changes in household responsibilities and pressures, and changes in the forms of domestic violence.

The developing literature on (mainly intimate partner) domestic violence during the Covid-19 pandemic identifies a number of important factors. One way to categorise them is according to the socio-ecologic model of violence against women proposed by Heise, which structures the influences on personal history, relationship and family, community, and society.[25] A review by Sánchez and colleagues usefully takes this approach to the effects of the pandemic.[9]

The overall picture develops from a combination of theory and empirical evidence. Lockdown or variants of it means that women are in enforced proximity to perpetrators of violence.[20 26 27] Isolated and unable to seek help,[14 28] they have fewer opportunities to report violence or to escape the situation.[1 4] Limited contact with other people and limited ability to go out exacerbate mental health concerns such as anxiety and depression and further reduce their willingness to seek care.[1 26] Their sources of social support are fewer than before,[1 9 28] and gender roles may lead to increased family obligations for female survivors. [14 1] Added to this is some reticence to seek healthcare or alternative shelter for fear of contracting Covid-19.[26] If women do seek care, service provision is itself compromised by closures.[1 9 29] Survivors looking for alternatives face constraints to communication by phone,[26] and surveillance of their social media and internet use.[4 28 30] Although online platforms that enable survivors to chat and access mobile apps to seek help have promoted access to support,[31] survivors isolating with family or their abusers or sharing their devices may not have privacy to safely use these platforms. They may also have little time for themselves due to the increase in responsibilities. The effects are exacerbated by the small living quarters common in urban informal settlements, making it even harder for survivors to find time alone. Perpetrators may also exploit the restrictions associated with the pandemic to increase their power and control over survivors,[14 30] including exploiting the fear of contagion.[4 9] Added distress from increased financial concerns, combined with the stress of confinement,[14 29] heightens aggression.[2] Perpetrators who drink alcohol may drink more at home because other venues are off limits.[9]

Our respondents largely confirmed these suppositions. There was, however, a different emphasis to their observations. All of them were already receiving support from our program. Although unacceptable, domestic violence was an existing feature of their lives on which the effects of lockdown played out. Some did say that confinement with their partner had led to an increase in physical violence, but their abiding concern was with finances and food security.[20] This was expected to some degree because of the hardships associated with poverty and informal settlements.[32 33] As Hamadani and colleagues have shown recently in rural Bangladesh, financial and food stress are accompanied by increases in violence.[34] The

For peer review only - http://bmjopen.bmj.com/site/about/guidelines.xhtml

second most common concern was with the burden of reproductive labour; notably, the pandemic's toll on women through the need for unpaid care and domestic work.[7 35] Gender and power analysis suggests that lockdown may affect the organisation of responsibilities, the roles of household members, and the organisation of power in the home, leading to abuse in response to structural threats.[33 36 37]

A third concern, and one that we have expressed before,[38] is that the global discussion tends to equate domestic violence with intimate partner violence. In our setting, although most physical and sexual violence is perpetrated by partners, around half of emotional violence is done by other family members. Lockdown may confine a woman with her husband, but it may equally confine her with her marital family, including members of the extended family who are living together for the duration. Around one-third of our clients have consulted because of intimate partner violence, around one-fifth because of family violence, and around one-quarter because of a combination of both. Emotional and economic violence are the reasons for consultation in most cases, although physical and sexual violence is common. The complexities of relationships mean that we should not leap to assumptions about domestic violence. Although many clients reported an increase in abuse, some said that the lockdown had improved their relationships because of time spent together, mutual respect, or close involvement in family life.

Two other issues deserve comment: alcohol and legal processes. Lack of availability of alcohol led in some cases to increased violence by habitual users. In others, it seems to have diminished irritability and allowed users to exhibit impulse control. Legal processes were to all intents and purposes halted during the pandemic,[8 11] increasing the stress on clients who were waiting for resolution of their cases.

Limitations

We do not know whether, at population level, the response to the pandemic increased the incidence or intensity of violence against women and girls. All our respondents had suffered physical, sexual, emotional, or economic violence and had previously reached a point at which they had consulted a counsellor. They were among the minority of survivors who had made the decision to share their concerns outside the home. This may partly explain the pragmatism that characterised their accounts of the situation during lockdown. They had already begun to think through and enact safety plans and coping strategies and violence was not their primary concern in this new and stressful environment.

Many of the factors influencing survivors' experiences, such as the reasons why some families enduring financial hardship would grow closer and some would experience more tension, are beyond our data. Methodological limitations include the fact that interviews relied on clients' access to phones and sufficient privacy to provide full and accurate details of their situation. Access to both of these is more limited during lockdown and may have affected the quality of answers given the increased presence of household members. Data quality and depth also varied between interviewers, who were collecting information within their counselling sessions.

Recommendations

It was and is difficult for policymakers and civil society organisations to respond to the needs of survivors of violence against women and girls during a pandemic. Our findings highlight the critical role of financial and social support, the need to factor domestic violence into response planning, and the importance of support networks.

Finance and food security are critical

Violence against women intersects with a lack of access to basic needs for money, food, and shelter. Any response to the current or future threat to public health must prioritise meeting these needs through economic and livelihood support,[29 34 39] simultaneously reducing the need to leave home to find food, rations, or work.[17 40] The two key sources of disadvantage

For peer review only

For peer review only - http://bmjopen.bmj.com/site/about/guidelines.xhtml

are poverty and gender. To address these sources, future strategies must include settlement community leaders in the design and implementation of pandemic management strategies and activities.

Domestic violence must be factored into response planning

Government and civil society must prioritise the continued provision of support for survivors of violence against women and girls.[7 34] Women need to know that help is available, how to get it, and that their abusers cannot take advantage of social changes to increase the frequency and intensity of violence.[7] This means that services for survivors of violence must be included in national preparedness, response, and recovery plans.[29 35] Responsive funding must be available and be deliverable to the third sector.[29] If programs are to provide more holistic health protection for survivors of domestic violence, they must involve them in planning. Awareness of the issue is paramount and the media should be enlisted (and step forward, as many have done).[29] Civil society organisations who are engaged in community outreach should continue and augment efforts to mobilise communities around intolerance of violence and include training programs on violence arising during natural disasters. Examples include training public health and law enforcement officials to understand and identify domestic violence,[41] community mobilisation motivated in part or whole by prevention of violence, and allocation of safe shelter for survivors at risk. The Covid-19 pandemic has highlighted the need for infrastructure in short-stay and shelter homes. Lack of space, unventilated rooms, and inadequate sanitation and hygiene facilities were major reasons for not being able to give shelter to women survivors during the pandemic. Alternative support networks are needed for women who are unable to safely remain at home, are evicted, are generally unable to support themselves, or are unsupported by their families.

Support networks are important

Our findings underline the central role of support networks that can identify survivors of violence and help them communicate with services.[7] It may be necessary for service providers to pivot to provide individual and group communication electronically rather than face-to-face. Civil society organisations could expand their remit in two areas. First, preventive counselling to promote healthy relationships and attitudes, beliefs, and behaviours among survivors and perpetrators, and foster problem-solving skills. Second, prevention programs that make communities more aware of their role in identifying and intervening in cases of intimate-partner and domestic violence. Current efforts to address domestic violence during the Covid-19 pandemic, including safe-word alert systems, digital or online communications and services, and remote counselling,[31 42] face challenges in our context. For example, a number of respondents were isolated by abusers who used lockdown to control their movement. This prevented them from accessing pharmacies and shops where a safe-word system is typically implemented.

CONCLUSION

In beginning to understand the impact of Covid-19 and subsequent lockdown measures on survivor needs and vulnerabilities, our findings underline the importance of financial precarity and access to resources and support. The response to the epidemic is an increase in existing stressors—poverty, gendered reproductive labour, anxiety about illness—as well as changes in households and patterns of alcohol use. Programs must focus on structural vulnerability, distributing resource-based support, prioritising the continued availability of services for survivors of violence, and expanding access to support networks. Decision-makers must be aware of the gendered and intersectional effects of interventions and must include survivors of domestic violence in the planning and implementation of public health strategies.

ACKNOWLEDGMENTS

For peer review only - http://bmjopen.bmj.com/site/about/guidelines.xhtml

We thank the counsellors who spoke with their clients and collected the information on which the paper was developed. We thank counselling coordinators Jyoti Borkar, Vandana Rokde, Vandana Singh, and Shirisha Yeotikar, counselling and legal coordinator Reshma Jagtap, and associate program director Sangeeta Punekar. Archana Bagra provided financial oversight and Vanessa D'Souza and Shanti Pantvaidya organisational leadership.

FUNDING STATEMENT

This work was done by the NIHR Global Health Research Group on a package of care for the mental health of survivors of violence in South Asia, funded by the UK National Institute for Health Research (17/63/47). The funder had no role in study design, data collection and analysis, decision to publish, or preparation of the manuscript.

Contributorship

ND and TD conceived the study. ND and DO acquired funding. TD and MH developed the methodology. ND, TD, DD, and DO oversaw investigation. MH, TD, and DO curated the data. MH did the first analysis and wrote the first draft. ND managed the project with support from TD. All authors critically reviewed drafts of the manuscript and read and commented on the final version. DO accepts full responsibility for the work and the conduct of the study, had access to the data, and controlled the decision to publish.

Competing interest

The authors declare no competing interest.

Data availability

Data are available in Open Science Framework: ***link will be provided on publication***

REFERENCES

1. Hall BJ, Tucker JD. Surviving in place: the coronavirus domestic violence syndemic. *Asian J Psychiatry* 2020;53:102179.
2. Mazza M, Marano G, Lai C, et al. Danger in danger: Interpersonal violence during COVID-19 quarantine. *Psychiatry Res* 2020;289:113046. doi: 10.1016/j.psychres.2020.113046 [published Online First: 2020/11/27]
3. Evans ML, Lindauer M, Farrell ME. A Pandemic within a Pandemic - Intimate Partner Violence during Covid-19. *N Engl J Med* 2020;383(24):2302-04. doi: 10.1056/NEJMp2024046 [published Online First: 2020/09/17]
4. Bradbury-Jones C, Isham L. The pandemic paradox: The consequences of COVID-19 on domestic violence. *J Clin Nurs* 2020;29(13-14):2047-49. doi: 10.1111/jocn.15296 [published Online First: 2020/04/14]
5. UN Women. The shadow pandemic: violence against women during COVID-19 2021 [Available from: https://www.unwomen.org/en/news/in-focus/in-focus-gender-equality-in-covid-19-response/violence-against-women-during-covid-19 accessed 8 Feb 2021.
6. Fraser E. Impact of COVID-19 pandemic on violence against women and girls. VAWG Helpdesk Research Report No 284. London: VAWG Helpdesk, 2020.
7. World Bank Group. Gender dimensions of the COVID-19 pandemic. Policy Notes. Washington DC: World Bank, 2020.
8. UN Women. Impact of COVID-19 on violence against women and girls and service provision: UN Women rapid assessment and findings. COVID-19 Response. New York NY: United Nations Women, 2020.
9. Sánchez OR, Vale DB, Rodrigues L, et al. Violence against women during the COVID-19 pandemic: An integrative review. *Int J Gynaecol Obstet* 2020;151(2):180-87. doi: 10.1002/ijgo.13365 [published Online First: 2020/09/04]
10. Agüero JM. COVID-19 and the rise of intimate partner violence. *World Dev* 2021;137:105217. doi: 10.1016/j.worlddev.2020.105217 [published Online First: 2020/10/06]

For peer review only - http://bmjopen.bmj.com/site/about/guidelines.xhtml

11. Majumdar S, Wood G. UNTF EVAW Briefing Note on the impact of COVID-19 on violence against women through the lens of Civil Society and Women's Rights Organizations. New York NY: UN Trust Fund to End Violence Against Women, 2020.
12. Gosangi B, Park H, Thomas R, et al. Exacerbation of Physical Intimate Partner Violence during COVID-19 Pandemic. *Radiology* 2021;298(1):E38-e45. doi: 10.1148/radiol.2020202866 [published Online First: 2020/08/14]
13. Evans DP. COVID-19 and violence: a research call to action. *BMC Womens Health* 2020;20(1):249. doi: 10.1186/s12905-020-01115-1 [published Online First: 2020/11/12]
14. Viero A, Barbara G, Montisci M, et al. Violence against women in the Covid-19 pandemic: A review of the literature and a call for shared strategies to tackle health and social emergencies. *Forensic Sci Int* 2020;319:110650. doi: 10.1016/j.forsciint.2020.110650 [published Online First: 2020/12/20]
15. WHO. COVID-19 and violence against women. What the health sector/system can do. Geneva: World Health Organization, 2020.
16. United Nations Human Settlements Programme (UN-Habitat). The challenge of slums: global report on human settlements 2003. London and Sterling VA: Earthscan Publications Ltd 2003.
17. Golechha M. COVID-19 Containment in Asia's Largest Urban Slum Dharavi-Mumbai, India: Lessons for Policymakers Globally. *J Urban Health* 2020;97(6):796-801. doi: 10.1007/s11524-020-00474-2 [published Online First: 2020/08/21]
18. Slater J, Masih N, Parth MN. In one of the world's largest slums, the fight against the coronavirus has turned into a struggle to survive. *Washington Post* 2020 12 May 2020.
19. Ray D, Subramanian S, Vandewalle L. India's lockdown. CEPR Policy Insight No 102. London: Centre for Economic Policy Research, 2020.
20. Vora M, Malathesh BC, Das S, et al. COVID-19 and domestic violence against women. *Asian J Psychiatr* 2020;53:102227-27. doi: 10.1016/j.ajp.2020.102227 [published Online First: 2020/06/15]
21. UN Women. COVID-19 and essential services provision for survivors of violence against women and girls. COVID-19 Response. New York NY: United Nations Women, 2020.
22. Chakraborty P, Daruwalla N, Jayaraman A, et al. "You Are a Part of the Solution": Negotiating Gender-Based Violence and Engendering Change in Urban Informal Settlements in Mumbai, India. *Violence Against Women* 2016 doi: 10.1177/1077801216659941 [published Online First: 2016/08/06]
23. Gale NK, Heath G, Cameron E, et al. Using the framework method for the analysis of qualitative data in multi-disciplinary health research. *BMC Med Res Methodol* 2013;13:117. doi: 10.1186/1471-2288-13-117 [published Online First: 2013/09/21]
24. Smith J, Firth J. Qualitative data analysis: the framework approach. *Nurse Researcher* 2011;18:52-62.
25. Heise L. Violence against women: an integrated ecological framework. *Violence Against Women* 1998;4:262-90.
26. Moreira DN, Pinto da Costa M. The impact of the Covid-19 pandemic in the precipitation of intimate partner violence. *Int J Law Psychiatry* 2020;71:101606. doi: 10.1016/j.ijlp.2020.101606 [published Online First: 2020/08/10]
27. Jarnecke AM, Flanagan JC. Staying safe during COVID-19: How a pandemic can escalate risk for intimate partner violence and what can be done to provide individuals with resources and support. *Psychol Trauma* 2020;12(S1):S202-s04. doi: 10.1037/tra0000688 [published Online First: 2020/06/20]
28. Campbell AM. An increasing risk of family violence during the Covid-19 pandemic: strengthening community collaborations to save lives. *Forensic Sci Int* 2020;Reports 2:100089.
29. UN Women. Prevention: violence against women and girls & COVID-19. COVID-19 Response. New York NY: United Nations Women, 2020.
30. Ragavan MI, Garcia R, Berger RP, et al. Supporting Intimate Partner Violence Survivors and Their Children During the COVID-19 Pandemic. *Pediatrics* 2020;146(3) doi: 10.1542/peds.2020-1276 [published Online First: 2020/08/21]

For peer review only

31. Emezue C. Digital or digitally delivered responses to domestic and intimate partner violence during COVID-19. *JMIR Public Health Surveill* 2020;6:e19831.
32. Al-Ali N. Covid-19 and feminism in the Global South: Challenges, initiatives and dilemmas. *European Journal of Women's Studies* 2020;27(4):333-47. doi: 10.1177/1350506820943617
33. Nigam S. COVID-19: India's response to domestic violence needs rethinking. *South Asia J* 2020;Available at SSRN: https://ssrn.com/abstract=3598999
34. Hamadani JD, Hasan MI, Baldi AJ, et al. Immediate impact of stay-at-home orders to control COVID-19 transmission on socioeconomic conditions, food insecurity, mental health, and intimate partner violence in Bangladeshi women and their families: an interrupted time series. *The Lancet Global health* 2020;8(11):e1380-e89. doi: 10.1016/s2214-109x(20)30366-1 [published Online First: 2020/08/29]
35. UN Women. From insights to action: gender equality in the wake of COVID-19. COVID-19 Response. New York NY: United Nations Women, 2020.
36. Savinaya HL. A study on domestic violence against women during COVID-19 lockdown in India. *Mukt Shabd J* 2020;9:1564-72.
37. Chalakkal K. Increase In Domestic Violence Against Women And Children During COVID-19. 2020
38. Daruwalla N, Kanougiya S, Gupta A, et al. Prevalence of domestic violence against women in informal settlements in Mumbai, India: a cross-sectional survey. *BMJ open* 2020;10(12):e042444. doi: 10.1136/bmjopen-2020-042444 [published Online First: 2020/12/18]
39. Béland L-P, Brodeur A, Haddad J, et al. COVID-19, family stress and domestic violence: remote work, isolation and bargaining power. IZA DP No 13332. Bonn: Institute of Labor Economics, 2020.
40. Chakraborty P. Distribution of fresh fruits and vegetables in Dharavi, India: lessons and learnings from the Covid-19 pandemic. Mumbai: Society for Nutrition, Education and Health Action, 2020.
41. Peterman A, Potts A, O'Donnell M, et al. Pandemics and violence against women and children. Working Paper 528. Washington DC: Center for Global Development, 2020.
42. Natarajan J. Women's safety during lockdown. *The Hindu* 2020 13 April 2020.

For peer review only

COREQ (COnsolidated criteria for REporting Qualitative research) Checklist

A checklist of items that should be included in reports of qualitative research. You must report the page number in your manuscript where you consider each of the items listed in this checklist. If you have not included this information, either revise your manuscript accordingly before submitting or note N/A.

Topic	Item No.	Guide Questions/Description	Reported on Page No.
**Domain 1: Research team and reflexivity**			
Personal characteristics			
Interviewer/facilitator	1	Which author/s conducted the interview or focus group?	5
Credentials	2	What were the researcher's credentials? E.g. PhD, MD	5
Occupation	3	What was their occupation at the time of the study?	5
Gender	4	Was the researcher male or female?	5
Experience and training	5	What experience or training did the researcher have?	5
Relationship with participants			
Relationship established	6	Was a relationship established prior to study commencement?	5
Participant knowledge of the interviewer	7	What did the participants know about the researcher? e.g. personal goals, reasons for doing the research	5
Interviewer characteristics	8	What characteristics were reported about the inter viewer/facilitator? e.g. Bias, assumptions, reasons and interests in the research topic	5
**Domain 2: Study design**			
Theoretical framework			
Methodological orientation and Theory	9	What methodological orientation was stated to underpin the study? e.g. grounded theory, discourse analysis, ethnography, phenomenology, content analysis	5
Participant selection			
Sampling	10	How were participants selected? e.g. purposive, convenience, consecutive, snowball	5
Method of approach	11	How were participants approached? e.g. face-to-face, telephone, mail, email	5
Sample size	12	How many participants were in the study?	6
Non-participation	13	How many people refused to participate or dropped out? Reasons?	6
Setting			
Setting of data collection	14	Where was the data collected? e.g. home, clinic, workplace	5
Presence of non-participants	15	Was anyone else present besides the participants and researchers?	5
Description of sample	16	What are the important characteristics of the sample? e.g. demographic data, date	6
Data collection			
Interview guide	17	Were questions, prompts, guides provided by the authors? Was it pilot tested?	5
Repeat interviews	18	Were repeat inter views carried out? If yes, how many?	0
Audio/visual recording	19	Did the research use audio or visual recording to collect the data?	0
Field notes	20	Were field notes made during and/or after the inter view or focus group?	5
Duration	21	What was the duration of the inter views or focus group?	5
Data saturation	22	Was data saturation discussed?	0
Transcripts returned	23	Were transcripts returned to participants for comment and/or	0

For peer review only

Topic	Item No.	Guide Questions/Description	Reported on Page No.
		correction?	
**Domain 3: analysis and findings**			
Data analysis			
Number of data coders	24	How many data coders coded the data?	5
Description of the coding tree	25	Did authors provide a description of the coding tree?	5
Derivation of themes	26	Were themes identified in advance or derived from the data?	5
Software	27	What software, if applicable, was used to manage the data?	5
Participant checking	28	Did participants provide feedback on the findings?	0
Reporting			
Quotations presented	29	Were participant quotations presented to illustrate the themes/findings? Was each quotation identified? e.g. participant number	6-8
Data and findings consistent	30	Was there consistency between the data presented and the findings?	6-8
Clarity of major themes	31	Were major themes clearly presented in the findings?	6-8
Clarity of minor themes	32	Is there a description of diverse cases or discussion of minor themes?	9

Developed from: Tong A, Sainsbury P, Craig J. Consolidated criteria for reporting qualitative research (COREQ): a 32-item checklist for interviews and focus groups. *International Journal for Quality in Health Care*. 2007. Volume 19, Number 6: pp. 349 – 357

Once you have completed this checklist, please save a copy and upload it as part of your submission. DO NOT include this checklist as part of the main manuscript document. It must be uploaded as a separate file.

For peer review only

BMJ Open

Intersectional tension: a qualitative study of the effects of the Covid-19 response on survivors of violence against women in urban India

Journal:	*BMJ Open*
Manuscript ID	bmjopen-2021-050381.R1
Article Type:	Original research
Date Submitted by the Author:	25-May-2021
Complete List of Authors:	Huq, Mita; UCL, Institute for Global Health<br>Das, Tanushree; Society for Nutrition, Education & Health Action, Program on Prevention of Violence Against Women and Children<br>Devakumar, Delan; University College London, Institute for Global Health<br>Daruwalla, Nayreen; Society for Nutrition, Education & Health Action, Program on Prevention of Violence Against Women and Children<br>Osrin, David; University College London, Institute for Global Health
**Primary Subject Heading**:	Public health
Secondary Subject Heading:	Global health
Keywords:	COVID-19, Public health < INFECTIOUS DISEASES, MENTAL HEALTH, QUALITATIVE RESEARCH

SCHOLARONE™
Manuscripts

I, the Submitting Author has the right to grant and does grant on behalf of all authors of the Work (as defined in the below author licence), an exclusive licence and/or a non-exclusive licence for contributions from authors who are: i) UK Crown employees; ii) where BMJ has agreed a CC-BY licence shall apply, and/or iii) in accordance with the terms applicable for US Federal Government officers or employees acting as part of their official duties; on a worldwide, perpetual, irrevocable, royalty-free basis to BMJ Publishing Group Ltd ("BMJ") its licensees and where the relevant Journal is co-owned by BMJ to the co-owners of the Journal, to publish the Work in this journal and any other BMJ products and to exploit all rights, as set out in our licence.

The Submitting Author accepts and understands that any supply made under these terms is made by BMJ to the Submitting Author unless you are acting as an employee on behalf of your employer or a postgraduate student of an affiliated institution which is paying any applicable article publishing charge ("APC") for Open Access articles. Where the Submitting Author wishes to make the Work available on an Open Access basis (and intends to pay the relevant APC), the terms of reuse of such Open Access shall be governed by a Creative Commons licence – details of these licences and which Creative Commons licence will apply to this Work are set out in our licence referred to above.

Other than as permitted in any relevant BMJ Author's Self Archiving Policies, I confirm this Work has not been accepted for publication elsewhere, is not being considered for publication elsewhere and does not duplicate material already published. I confirm all authors consent to publication of this Work and authorise the granting of this licence.

For peer review only

Intersectional tension: a qualitative study of the effects of the Covid-19 response on survivors of violence against women in urban India

AUTHORS

Mita Huq

Institute for Global Health, University College London, London, WC1N IEH, UK

mita.huq.19@ucl.ac.uk

Tanushree Das

Program on Prevention of Violence Against Women and Children, SNEHA, Mumbai, Maharashtra, 400017, India

tanushree.das@snehamumbai.org

Delan Devakumar

Institute for Global Health, University College London, London, WC1N IEH, UK

d.devakumar@ucl.ac.uk

Nayreen Daruwalla

Program on Prevention of Violence Against Women and Children, SNEHA, Mumbai, Maharashtra, 400017, India

nayreen@snehamumbai.org

David Osrin (**corresponding author)**

Institute for Global Health, University College London, London, WC1N IEH, UK

d.osrin@ucl.ac.uk

For peer review only - http://bmjopen.bmj.com/site/about/guidelines.xhtml

ABSTRACT

Objectives

There is concern worldwide that the physical and emotional constraints that arise from efforts to address the SARS-CoV-2 pandemic have affected the frequency and intensity of domestic violence against women. Residents of urban informal settlements faced particularly stringent conditions during the response in India. Counsellors spoke with registered survivors of domestic violence in Mumbai, with two objectives: to understand how the pandemic and subsequent lockdown had changed their needs and experiences, and to recommend programmatic responses.

Design

Qualitative interviews and framework analysis.

Setting

A non-government support program for survivors of violence against women, providing services mainly for residents of informal settlements.

Participants

During telephone follow-up with registered survivors of violence against women with whom they already had a relationship, counsellors asked about the effects of Covid-19 on their daily and family life, their ability to speak with someone, and their counselling preferences.

Results

The major concerns of 586 clients interviewed between 1st April and 30th July 2020 were meeting basic needs (financial stress, interrupted livelihoods, and food insecurity), confinement in small homes (family tensions and isolation with abusers), and limited mobility (power imbalances in the home and lack of opportunity for disclosure and stress relief). A major source of stress was the increased burden of unpaid domestic care, which fell largely on women and triggered resentment and conflict.

Conclusion

The Covid-19 pandemic has increased the burden of poverty and gendered unpaid care. Finance and food security are critical considerations for future response, which should consider inequality, financial support, prioritising the continued availability of services for survivors of violence, and expanding access to social networks. Decision-makers must be aware of the gendered and intersectional effects of interventions and must include residents of informal settlements who are survivors of domestic violence in the planning and implementation of public health strategies.

KEYWORDS

Covid-19; Domestic Violence; Gender-based violence; poverty areas; Mumbai; India

For peer review only

For peer review only - http://bmjopen.bmj.com/site/about/guidelines.xhtml

Article summary

Strengths and limitations of this study

Strengths

- Interviews were conducted with almost 600 survivors of violence.
- Existing relationships between survivors and counsellors.
- All participants were residents of urban informal settlements in which stringent measures to limit the spread of Covid-19 were implemented.

Limitations

- The study did not examine incidence of domestic violence against women who were not already registered.
- Telephone interviews were limited by women's willingness to provide information.

For peer review only

BACKGROUND

As governments implemented measures to address the spread of SARS-CoV-2, organisations working to prevent violence against women became concerned that the measures might themselves give rise to or escalate domestic violence.[1-5] This concern was informed by three strands of evidence. The first was that states and civil society organisations noted large increases in contacts through domestic violence helplines and websites at the same time as the numbers of face-to-face consultations fell.[6-13] The second was that the conditions imposed by the pandemic, such as lockdown and income loss, were likely to increase household and interpersonal conflict.[14 15] The third was that public health emergencies have been shown repeatedly to be followed by increases in violence against women and girls.[16 17] Amid reports that the frequency and severity of domestic violence were increasing,[17-20] the World Health Organization (WHO) and United Nations Women (UN Women) raised concerns that measures such as quarantine and lockdown could increase the risk of violence against women.[8 21]

These concerns were observable in India, where an increase in violence against women was recognised in the media and some studies.[22 23] The reports describe a relationship between loss of economic prospects, separation from friends and family, and amplified gendered expectations as a result of lockdown measures. Lockdown measures were implemented suddenly and stringently, particularly in urban informal settlements (slums). Residents of such settlements, in which ~40% of Mumbai's households are located, were seen as hotspots for contagion because of density and overcrowding, insubstantial housing, and insufficient water and sanitation.[24 25] An average of five people share one or two rooms, most rely on shared toilets, and around two-thirds need to leave the house to get water. The government of Maharashtra announced a lockdown in Mumbai on 23rd March 2020, for an initial 21 days with subsequent extensions. Major informal settlements were quarantined as red zones,[26] their access barricaded, public toilets disinfected, and a police-enforced curfew instituted. Residents were confined to their homes unless they faced medical emergencies, transport services and vehicular movement curtailed, and industry and retail outlets closed. Residents were screened for SARS-CoV-2 through door-to-door visits by public and private sector healthcare workers and centres providing free food and healthcare were set up in schools and halls.[25]

These measures were commended for preventing a surge in cases in areas where the potential spread of SARS-CoV-2 was particularly concerning,[25 27] but fear of institutional quarantine was common and accompanied by a degree of community stigma. The risk of domestic violence does not appear to have been considered. At the same time as survivors of violence were potentially confined with their abusers, organisations working to prevent and respond to violence suffered resource limitations,[28 29] reduced capacities, and interrupted monitoring and evaluation activities.[8 21 30] The civil society work of SNEHA (Society for Nutrition, Education and Health Action) illustrates these pressures. Since 2000, the SNEHA Program on Prevention of Violence Against Women and Children has provided support to over 13 000 survivors of violence against women. Services are open to all—particularly residents of Mumbai's informal settlements—and are delivered from five community and three public hospital counselling centres. Support is available through a combination of in-house services and liaison. In-house human resources include community outreach workers, counsellors, legal advisors and lawyers, and psychologists. These are complemented by liaison with hospital practitioners, the police, District Legal Aid services, shelters, and psychiatrists.

As the response to the pandemic unfolded, the programme moved counselling and crisis intervention services online. Existing helplines were augmented and survivors of violence could call or email counselling centres for assistance. A cadre of 300 volunteers gave out contact details in communities. At the same time, counsellors contacted their existing clients and offered to provide follow-up telephonically. SNEHA developed a protocol for this follow-up, based on existing safety protocols and emphasising confidentiality and the same client-

For peer review only - http://bmjopen.bmj.com/site/about/guidelines.xhtml

centred approach followed in face-to-face sessions. It became clear that these telephone conversations could provide a rich understanding of women's experiences of domestic violence in urban informal settlements during lockdown. In discussion with counsellors, we designed a topic guide for qualitative interviews that they could integrate into their discussion with existing clients. Our objectives were to use the interviews to understand how the Covid-19 pandemic and subsequent lockdown changed women's needs and experiences, and to recommend programmatic responses to domestic violence in light of pandemics and the strategies used to mitigate them.

METHODS

Setting and participants

Our study explored the experiences of survivors of domestic violence in a specific context:[31] residents of urban informal settlements in Mumbai whose suffering as a consequence of domestic violence by either an intimate partner or another family member had led them to register for support. Only about 8% of women in these circumstances get as far as registering with a support organisation,[32] and the sample therefore represents a selected group. Interviewees were women clients who had been interacting with SNEHA counsellors prior to the lockdown and had developed relationships with them. They were all survivors of violence, having connected with the organisation after surviving domestic violence—physical, sexual, emotional, or economic violence, coercive control or neglect—by intimate partners or family members. Since they were all survivors, our interest was in changes in their experience rather than in the incidence of violence against women who had not previously made contact.

Data collection

Eleven female counsellors with postgraduate education in social work or psychology and between two and ten years of experience of supporting clients from our centres conducted interviews after receiving project-specific training. Clients had registered initially through self-report, referral from agencies such as the police, clinicians, or non-government organizations, or referral by SNEHA community organisers or volunteers trained to identify and engage with survivors. Participants were contacted by their counsellors for telephone follow-up between March and May 2020. Counsellors explained that, as part of their conversations, they were doing a study to understand women's experiences during the lockdown. With agreement from clients, they administered a semi-structured interview about how Covid-19 had affected their daily life, family life, ability to speak with someone, and their counselling preferences. Interviews were conducted during working hours. If a client did not answer the phone, nearby field staff, volunteers, or women's group members would visit her and encourage her to contact her counsellor to indicate her availability. Counsellors provided all clients with information on Covid-19 (transmission, social distancing, handwashing, masks, emotional hygiene, anxiety management, freedom to contact the organisation for advice). If a family member answered the phone (or it seemed likely to the counsellor that someone was listening in), they said that the call was part of the Covid-19 prevention program and that they were interested in the family's emotional health. Counsellors recorded responses in field notes categorised according to the topic guide. Interviews were conducted in Hindi and Marathi and subsequently summarised in English by bilingual team leaders. Interviews were part of counselling calls that lasted up to an hour. All potential identifiers were removed from the dataset.

Data analysis

The familiarisation process began by reading all the records. Two authors approached the data independently (MH and DO). We used the framework approach to thematic analysis.[33] Increasingly used in health research,[34] this approach lends itself to studies by multidisciplinary teams with fairly specific objectives and questions, limited time for data collection, and a predefined large sample.[35] Data are charted in a matrix in which rows

For peer review only - http://bmjopen.bmj.com/site/about/guidelines.xhtml

represent interviewees and columns represent codes, and information can be compared across cases and codes. We identified some of these codes before the analysis and augmented them with codes that emerged.[33 36-39] The two authors who analysed the data compared their codes iteratively. The emergent themes were discussed over four iterations, with inputs from all authors. An indexing and data exploration process expanded this framework by inductively adding themes and sub-themes.

Patient and public involvement

Clients of our counselling centres for survivors of violence against women were first involved during telephonic discussions with counsellors. The research questions were a direct response to concerns about their safety and emotional health and wellbeing during the response to the Covid-19 pandemic, and sought to understand their experiences and preferences for services. Dissemination will involve personal feedback of the findings to clients during subsequent counselling sessions and feedback during regular community mobilisation meetings in informal settlements across the city.

RESULTS

Participants

Between 1st April and 30th July 2020, counsellors spoke with 720 clients. Our analysis draws on interviews with 586 women for whom complete background information was available. At the time of interview, 54% of respondents were in the first month of lockdown, 40% in the second, and 6% in the third month or later. Of the existing clients who provided information, 46% had been seeing their counsellor for less than six months, 33% for up to a year, 12% for up to 18 months, and 8% for longer periods. Table 1 shows that most women were in their 20s or 30s (80%) and had attended school to at least secondary level (74%). Most had been married at some point (92%) and around two-thirds had children (63%). The largest group had been living in nuclear families at the time of initial consultation (59%) and the next largest group in joint families with their in-laws (33%). Most lived in informal settlements (*zopadpatti*) or former workers' accommodation (*chawls*) (73%). The major religions were about equally represented. Around one-third of women (32%) worked outside the home, usually in the informal sector.

The background to initial consultation for most women had been intimate partner violence (70%). However, 44% had suffered violence by another family member and in 28% of cases violence came from both intimate partners and other family members. Family violence was predominantly emotional (87%), economic (63%), and physical (54%), and coercive control (46%). The primary concerns that women said had led them to consult were economic abuse, particularly feeling that they had been denied money (56%), physical violence (49%), being compelled to leave their home (32%), being threatened by their partner (25%), having had their property or possessions taken by family members (19%), and suffering sexual violence (17%). Although instances of violence were not always the triggers for consultation, 86% of women described experiences of emotional violence, 76% economic violence, 64% physical violence, 56% neglect, 56% controlling behaviours, and 35% sexual violence. Most had survived more than one of these forms of violence (86%) and 22% all six forms.

For peer review only - http://bmjopen.bmj.com/site/about/guidelines.xhtml

Table 1. Participant characteristics at registration for counselling

Age (years)	n	(%)
Under 20	26	(4)
20-29	262	(45)
30-39	205	(35)
40-49	56	(10)
50+	37	(6)
**Education**		
None	63	(11)
Primary	78	(13)
Lower secondary	97	(16)
Higher secondary	226	(39)
Higher	110	(19)
Unknown	12	(2)
**Marital status**		
Married	431	(73)
De facto	16	(3)
Separated or divorced	64	(11)
Widowed	29	(5)
Unmarried	45	(8)
**Children**		
None	217	(37)
1	149	(25)
2 or more	220	(38)
**Family arrangement**		
Nuclear	342	(59)
Joint	194	(33)
Extended	23	(4)
Living alone	19	(3)
Other	8	(1)
**Religion**		
Hindu	278	(47)
Muslim	241	(41)
Other	67	(12)
**Remunerated employment**		
None	392	(67)
Student	7	(1)
Informal sector	127	(22)
Formal sector	60	(10)
All	586	(100)

A matrix of stressors

What emerged from women's accounts of life during lockdown was a matrix of stressors that interacted with domestic violence: worry about basic needs, confinement in small homes, limited mobility, concern for children, and alcohol. Lockdown had confined them to four general living arrangements: a nuclear family, a joint family with husband and in-laws, with members of a natal family, or either alone or with children. At the time of interview, around half of respondents (52%) were living with their partner in either nuclear or joint families. Most women who were not had relocated to live with their natal family. This usually represented a longer-term strategy of separation, but for some a short visit had become longer as a result of lockdown. For women who were confined in a nuclear family with a husband who had been abusive at the time of initial consultation, 27% were still surviving

For peer review only - http://bmjopen.bmj.com/site/about/guidelines.xhtml

For peer review only

violence. For women who were living with a husband and in-laws, 28% were still surviving intimate partner or family violence.

Worry about basic needs

Dominant in women's narratives were problems meeting basic needs. Half of respondents expressed overwhelming concerns about money and food, and most women who said that they were managing were drawing on their own or their family's savings. Families living in informal settlements depend largely on a mosaic of livelihoods in the informal sector and most women said that the lockdown had caused work to be suspended. Restrictions on movement meant that daily earners could not go to work: “I used to do domestic work but am now unable to go to work. My employers have been calling me to say that because … Covid-19 cases are high here, they don't want me to join work for another six months. I'm worried about finances” (20-29 years, separated with 1 child and living with natal family; physical, emotional, and economic IPV at first consultation).

A few days of lockdown led many families to draw on their limited savings. “We're running out of savings. My husband's boss is not able to pay him right now but is providing him with the basics so he's staying at the job. But my kids and I are struggling to make ends meet” (30-39 years, married with 4 children and living with husband in nuclear family; physical, emotional, economic IPV). The only safety net was the government ration system, itself compromised by halts in supply and quarantine of communities. Families who were eligible for rations became more dependent on them, but the quantity and quality of rations and food in the shops was compromised by supply problems. “The food resources have been insufficient, as well as the whole family is present and two meals for all become a little tough with this current crisis” (20-29 years, unmarried and living in natal family; physical IPV). Food insecurity therefore resulted from both lack of money and unavailability of food: “All shops are closed. The area is sealed. We are managing with the rations we get from the ration shop. Other shops are closed. Around 15 days back a lady had come to ask for our contact numbers to provide help, but we haven't heard from her or anyone so far” (20-29 years, unmarried and living in natal family; physical family violence). Coupled with inconsistent or insufficient government and non-government assistance, these pressures made members of food insecure families leave the home in contravention of lockdown policy to seek food, exposing themselves to SARS-CoV-2 and punitive action by law enforcement. The combination of insecurity and violence was a potent stressor: “Barely being able to manage. My husband came and fought with me a lot a couple of days ago. After that now the shop below my house is giving me stuff on loan” (20-29 years, married with 3 children and living with children; physical, sexual, emotional, economic IPV).

Confinement in small homes

Stress was increased by the small living spaces in informal settlements in which most respondents lived. Lack of privacy and time alone exacerbated tension in large families, whereas loneliness was reported by women who lived alone or were being forcibly isolated or neglected. Tension was often increased by a combination of overcrowding and precarity. “… I'm in tension and stress due to lack of money and resources. Sometimes we fight with each other, but … I'm not able to go outside…” (40-49 years, married with 6 children and living with husband in nuclear family; physical, sexual, emotional, economic IPV). Women living in joint families faced predominantly emotional violence, and physical violence in three cases.

Proximity could, however, lead to improvements. For women living with abusive husbands, previously acrimonious relations sometimes improved: “… My husband came closer to me and my daughter, taking care of both of us” (30-39 years, married with 1 child and living with husband in nuclear family; physical, emotional, economic IPV). It was also possible for violence to decrease because of wariness rather than benevolence, when the abuser relied on the respondent: “My husband fights with me all the time. He constantly bickers. He's not beating me right now because he knows he can't do without me for now” (30-39 years,

For peer review only - http://bmjopen.bmj.com/site/about/guidelines.xhtml

married with 3 children and living with husband in joint family; physical, sexual, emotional, economic IPV and family violence).

Nevertheless, most women living with their natal family—away from the source of violence—were positive about it: “I love taking care of my family and being with them. Glad they support me so much. We're very happy we're getting quality time together” (30-39 years, separated with no children and living with natal family; physical, sexual, emotional, economic IPV).

Limited mobility

The effects of precarity and confinement were exacerbated by the limits set to mobility. These had two general effects: manifestation of power imbalances and an inability to find relief outside the home. For some women, confinement had “… given my husband more power. It's very difficult for us” (30-39 years, married with 4 children and living with husband in nuclear family; physical, sexual, emotional, economic IPV). Money was withheld or taken from women and abusers controlled their movements indoors, forced them out of the house, or isolated them socially. “My husband and in-laws are very abusive. Many times they have locked me up and left me without food” (20-29 years, married with no children and living with husband in joint family; physical, sexual, emotional, economic IPV and family violence). A few survivors said that their partners had not allowed them to access medical care on the pretext of acquiring infection.

Confinement also led to feelings of helplessness and lack of opportunity to decompress. Of the 208 women who had not reported physical violence at first consultation, four said that it had begun during the lockdown: “My husband beat me for the first time… My husband is getting violent and I'm alone at home and neither of us can leave to cool down. I feel trapped” (20-29 years, married with no children and living with husband in nuclear family; emotional IPV and family violence). Threats of violence from their abusers prevented women from disclosing their unhappiness. This was particularly concerning for respondents in abusive situations in need of counselling services, where the abuser was always around or monitoring their calls. Counsellors sometimes noted reticence in women’s answers to questions: “Client's husband was there and so she was being a little calculating about what she was saying” (20-29 years, married with 3 children and living with husband in nuclear family; physical, sexual, emotional, economic IPV). Women were unable to draw on emotional support outside the home from neighbours, friends, or family members. “Before this situation, I spent some time with my neighbours to share my feelings and thoughts, but now due to more housework, I’m not able to manage more time for myself” (30-39 years, married with 4 children and living with husband in nuclear family; physical, emotional, economic IPV and family violence). Women described hesitancy to disclose their emotions to others for fear of being judged or misunderstood, or because they had limited access to phones. They often felt that it was their responsibility to handle their mental health since the pandemic had already brought enough stress and fear to those around them. “I don't share with anyone. I don't want to trouble them. I don't talk to my family and I don't have any friends here. So I keep everything to me” (20-29 years, separated with 1 child and living with natal family; physical, sexual, emotional, economic IPV and family violence).

Concern for children

Beside their concern for their children’s diet, education, and socialisation, women were worried that fights, harassment, or physical violence that would otherwise have occurred while children were at school or outside the home now happened in front of them. One woman’s husband was “…staying at home the whole day … and that has been affecting the mental health of my kids also as they are afraid of the rage that they see after their father is drunk and acts aggressive or hits me” (30-39 years, married with 3 children and living with husband in nuclear family; physical, sexual, emotional, economic IPV).

Alcohol

For peer review only - http://bmjopen.bmj.com/site/about/guidelines.xhtml

Lockdown changed the availability of alcohol, leading to improvements in some households, but more drinking at home or withdrawal symptoms in others. “He has been creating a lot of issues being drunk all day and also forces me to have sex sometimes” (30-39 years, living with partner with 2 children in nuclear family; physical, sexual, emotional, economic IPV). For those reporting improvement, it was primarily through less drinking and more time spent caring for the family. “My husband has stopped drinking so there is some peace at home” (20-29 years, married with 3 children in nuclear family; physical, sexual, emotional, economic IPV). In some homes where drinking persisted, not only were tensions heightened, but abusers would leave the house to find alcohol. This caused further worries about exposure to SARS-CoV-2 and added to household fears for health and wellness.

“Women’s work”

Along with their concerns about basic needs, many women said that their main source of stress was the increase in household responsibilities that fell disproportionately on them. Some of these related to hygiene: cleaning, disinfection, and childcare in an environment in which viral transmission was a major concern. The extra housework occasioned by more people in the household and children being off school was usually shouldered by women. “I have extra workload and no help, while if I expect help my husband and mother-in-law abuse me” (30-39 years, married with 2 children and living with husband in joint family; physical, emotional, economic IPV). Women tended to be tasked with cooking and cleaning and men were tasked with leaving the house to get supplies. The cognitive effects of these responsibilities centred on weariness and frustration with gender norms, when families were “… using the lockdown for harassment and intimidation” (20-29 years, married with no children and living with husband in joint family; physical, sexual emotional, economic IPV and family violence).

Respondents who also worked from home reported difficulty balancing employment with their caring responsibilities, saying that family members had heightened expectations that they would complete chores. “I have to work more than before and my sister-in-law keeps saying that if I don’t work I am just another baggage using their resources” (20-29 years, married with no children and living with husband in joint family; physical, emotional, economic IPV). For these households, domestic labour ranked higher than work and school, and violating expectations could trigger abuse: “My natal family does emotional violence on me because I work from home, so the full day I am spending time at home, my family expects me to do domestic work” (20-29 years, divorced with 1 child and in joint family; emotional family violence). Even respondents who had moved in with their natal family said that they felt guilty for burdening them as they were unable to contribute financially, and that their presence added to precarity. “I am staying with my natal family and now that I am unable to contribute … I feel awkward and a burden to them. Not that they ever verbalized it, but the non-verbal gestures are enough to understand” (40-49 years, married with no children and living in natal family; physical, emotional, economic family violence).

Addressing needs with survivor-centred approaches

Respondents highlighted several needs during their interviews, many of them being basic survival supplies such as rations and money. Others flagged the need for legal counsel and continued court proceedings to address their domestic violence cases, all of which were interrupted by lockdown. A few needed medical attention and some simply wanted to see their friends and families or take a walk as a moment of solace.

They also discussed their experience with tele-counselling. Since all the respondents were survivors of violence with prior connections to counsellors, we asked how tele-counselling fit with their circumstances before and during the pandemic. Respondents recognised the importance of distanced counselling services during the pandemic, and generally found telephone discussion a good option. Some actually preferred it to in-person meetings, saying that it overcame geographic and physical barriers and time constraints that had prevented them from visiting a centre. Nevertheless, most said that they would prefer to return to the

For peer review only - http://bmjopen.bmj.com/site/about/guidelines.xhtml

counselling centre when it was safe. Respondents who reported substantial changes to their daily life said that they were unable to talk at home because of lack of privacy, family members listening in, higher risk of violence if discovered, or limited access to a phone. They said that in-person counselling was better because they felt more comfortable and articulate at the counselling centre and the services would be better. Respondents who did not report increased violence agreed with this, but needed different types of services from the centre, such as psychological counselling for family members other than themselves or legal guidance.

DISCUSSION

During the response to the Covid-19 pandemic, survivors of domestic violence in Mumbai's urban informal settlements faced—as well as fears of contagion—heightened financial and food insecurity, confinement in small homes in the presence or absence of abusers, coercive control, inability to discuss their experiences with others, and an increased burden of domestic labour. Legal processes were to all intents and purposes halted during the pandemic,[8 18] increasing the stress on clients who were waiting for resolution of their cases. Our study explored the needs and priorities of women whose relationships were already marked by intimate partner and family violence, and whose living conditions were already difficult. Despite seeking assistance from our counsellors, they lived in a state of constant fear about finances and food, an increased burden of household responsibilities, and tolerance of coercive behaviours.

Intersectional insecurity

Quarantine measures and restrictions on movement increased financial and food insecurity, linking to pathways of risk via economic constraint and exploitative relationships. All the respondents were already receiving support from our program. Although unacceptable, domestic violence was an existing feature of their lives on which the effects of lockdown played out. For some, confinement with their partner had led to an increase in physical violence, but their abiding concern was with finances and food security,[22] driven predominantly by loss of employment for either themselves or their family. The matrix of stressors echoes the concerns of intersectional feminism, which encourages broader consideration of social factors that shape survivorship.[40] The increased risk of violence in conditions in which the hardships associated with poverty and informal settlements are augmented is evident in India,[41] rural Bangladesh,[42] and in other lower-income countries.[43] We need to pay attention to survivors' basic needs: while some may benefit from exit strategies and social and legal protective services, there is a clear need for food and financial support.[14] Lack of availability of alcohol (or an increase in drinking at home)[9] led in some cases to increased violence by habitual users. In others, it seems to have diminished irritability and allowed users to exhibit impulse control.[28 29]

Confinement

Insecurity combines with enforced proximity with perpetrators of violence,[14 20 22 23 44 45] in small homes in urban informal settlements, amplifying the tension between family members and instances of domestic violence.[46] The global discussion tends to equate domestic violence with intimate partner violence, but violence by other family members is common in India. We have reported previously that other family members are responsible for around half of emotional violence against women in informal settlements in Mumbai.[32] Lockdown may confine a woman with her husband, but it may equally confine her with her marital family, including members of the extended family who are living together for the duration. This is exacerbated when perpetrators exploit the restrictions associated with the pandemic to exercise power and control,[6 47] including exploiting the fear of contagion.[4 9] Changes in household composition as a result of lockdown altered the situation in which violence occurred. This is an example of how social structure allows (or does not allow) people to translate criminal intentions into action. Routine activity theory suggests that an offence requires an offender, a suitable target, and absence of capable guardians, and it is

For peer review only - http://bmjopen.bmj.com/site/about/guidelines.xhtml

interesting to think about the effects of changes in who is present in the home as changes in a micro-ecology in which domestic violence takes place.[48]

Limited mobility and contact with other people contribute to mental health concerns such as anxiety and depression and further reduce willingness to seek care.[1 44] Lockdown led to decreased freedom and privacy, with attendant physical and psychological stress and fewer sources of social support.[1 9 49] Isolated and unable to seek help,[6 49] women had fewer opportunities to report violence or to escape the situation.[1 4] Although online platforms and mobile applications that enable survivors to chat and seek help have promoted access to support,[50] survivors isolating with family or sharing their devices face constraints to communication by phone,[44] and surveillance of their social media and internet use.[4 47 49] They may also have little time for themselves due to the increase in responsibilities and the small homes common in urban informal settlements. Added to this is some reticence to seek healthcare or alternative shelter for fear of contracting Covid-19.[44] If women do seek care, service provision is itself compromised by closures.[1 9 51]

Burden of unpaid care

The intersection between women's unpaid care work and violence, underpinned by social norms, has recently been highlighted in India.[52] Gender roles pervade experiences of domestic violence,[7 53 54] predominantly by way of family and social expectations. Sexism organises power in families,[55] the Covid-19 pandemic has increased the need for unpaid care and domestic work,[1 6 17 56] and women's perceived failure to meet the increased family obligations has resulted in abuse.[14]

Feminist political economy suggests that these outcomes were predictable: gendered division of labour in private and public domains define how women can earn a living.[57] The expectation that women perform unpaid domestic labour and that their access to paid work and education is undervalued sets the scene for the increased burden and sources of disagreement over the balance between domestic and remunerated work reported in our study. For many of our clients, the level of housework (often falling entirely on wives and daughters) increased, making it more difficult to complete. Some households divided duties such that women were to remain home while men left for supplies,[58] barring these women from accessing public channels for survivor support amidst an already diminished support landscape.[1 4 9 28] Added to this is the disproportionate effect of the pandemic on opportunities for women and girls, with drastic reductions in women's sources of work in agriculture and the informal sector reported in Nepal and Pakistan.[7 53]

In addition to their immediate health and safety, the needs born of these circumstances also cause concern for the long-term impacts on gender equality, particularly in terms of women's and girls' access to education and independence. This is illustrated by the rapidity with which lockdown returned women and girls to extended family contexts and the prioritising of housework above schoolwork and remunerated work.[59] Pressure to marry and depletion of work opportunities threaten women's educational prospects and their ability to sustain themselves independently.[29 53]

Limitations

We do not know whether, at population level, the response to the pandemic increased the incidence or intensity of violence against women and girls. All our respondents had suffered physical, sexual, emotional, or economic violence and had previously reached a point at which they had consulted a counsellor. They were among the minority of survivors who had made the decision to share their concerns outside the home. This may partly explain the pragmatism that characterised their accounts of the situation during lockdown. They had already begun to think through and enact safety plans and coping strategies and violence was not their primary concern in this new and stressful environment.

Many of the factors influencing survivors' experiences, such as the reasons why some families enduring financial hardship would grow closer and some would experience more

For peer review only - http://bmjopen.bmj.com/site/about/guidelines.xhtml

tension, are beyond our data. Methodological limitations include the fact that interviews relied on clients' access to phones and sufficient privacy to provide full and accurate details of their situation. Access to both of these is more limited during lockdown and may have affected the quality of answers given the increased presence of household members. Data quality and depth also varied between interviewers, who were collecting information within their counselling sessions.

Recommendations

It was and is difficult for policymakers and civil society organisations to respond to the needs of survivors of violence against women and girls during a pandemic. Our findings highlight the critical role of financial and social support, the need to factor domestic violence into response planning, and the importance of support networks.

Finance and food security are critical

Violence against women intersects with a lack of access to money, food,[60] and (for some) shelter. Any response to the current or future threat to public health must prioritise meeting these needs through economic and livelihood support,[42 51 61] simultaneously reducing the need to leave home to find food, rations, or work.[25 62] The two key sources of disadvantage are poverty and gender. To address these sources, future strategies must include community leaders in the design and implementation of pandemic management strategies and activities.

Support networks are important

Our findings underline the central role of support networks that can identify survivors of violence and help them communicate with services.[17] It may be necessary for service providers to pivot to provide individual and group communication electronically rather than face-to-face. Civil society organisations could expand their remit in two areas. First, preventive counselling to promote healthy relationships and attitudes, beliefs, and behaviours among survivors and perpetrators, and foster problem-solving skills. Second, prevention programs that make communities more aware of their role in identifying and intervening in cases of intimate-partner and domestic violence. It is especially important to take an approach that addresses harmful gendered norms and expectations due to the nature of violence in this context.[52] Current efforts to address domestic violence during the Covid-19 pandemic, including safe-word alert systems, digital or online communications and services, and remote counselling,[50 63] face challenges in our context. For example, a number of respondents were isolated by abusers who used lockdown to control their movement. This prevented them from accessing pharmacies and shops where a safe-word system is typically implemented.

Domestic violence must be factored into response planning

Government and civil society must prioritise the continued provision of support for survivors of violence against women and girls.[17 42] Women need to know that help is available, how to get it, and that their abusers cannot take advantage of social changes to increase the frequency and intensity of violence.[17] This means that services for survivors of violence must be included in national preparedness, response, and recovery plans.[51 56] Responsive funding must be available and be deliverable to the third sector.[51] If programs are to provide more holistic health protection for survivors of domestic violence, they must involve them in planning. Awareness of the issue is paramount and the media should be enlisted (and step forward, as many have done).[51] Civil society organisations who are engaged in community outreach should continue and augment efforts to mobilise communities around intolerance of violence and include training programs on violence arising during natural disasters. Examples include training public health and law enforcement officials to understand and identify domestic violence,[64] community mobilisation motivated in part or whole by prevention of violence, and allocation of safe shelter for survivors at risk.

For peer review only - http://bmjopen.bmj.com/site/about/guidelines.xhtml

CONCLUSION

In beginning to understand the impact of Covid-19 and subsequent lockdown measures on survivor needs and vulnerabilities, our findings underline the importance of financial precarity and access to resources and support. The response to the epidemic is an increase in existing stressors—particularly poverty and gendered household labour—as well as changes in households and patterns of alcohol use. Programs must focus on structural vulnerability, distributing resource-based support, prioritising the continued availability of services for survivors of violence, and expanding access to support networks. Decision-makers must be aware of the gendered and intersectional effects of interventions and must include survivors of domestic violence in the planning and implementation of public health strategies.

ACKNOWLEDGMENTS

We thank the counsellors who spoke with their clients and collected the information on which the paper was developed. We thank counselling coordinators Jyoti Borkar, Vandana Rokde, Vandana Singh, and Shirisha Yeotikar, counselling and legal coordinator Reshma Jagtap, and associate program director Sangeeta Punekar. Archana Bagra provided financial oversight and Vanessa D'Souza and Shanti Pantvaidya organisational leadership.

FUNDING STATEMENT

This work was done by the NIHR Global Health Research Group on a package of care for the mental health of survivors of violence in South Asia, funded by the UK National Institute for Health Research (17/63/47). The funder had no role in study design, data collection and analysis, decision to publish, or preparation of the manuscript.

Contributorship

ND and TD conceived the study. ND and DO acquired funding. TD and MH developed the methodology. ND, TD, DD, and DO oversaw investigation. MH, TD, and DO curated the data. MH did the first analysis and wrote the first draft. ND managed the project with support from TD. All authors critically reviewed drafts of the manuscript and read and commented on the final version. DO accepts full responsibility for the work and the conduct of the study, had access to the data, and controlled the decision to publish.

Competing interests

ND and TD work with SNEHA, the organisation at which the study was done.

Data availability

Data are available in Open Science Framework: ***link will be published on acceptance***

Ethics statement

The project was conducted within the NIHR Global Health Research Group on *A package of care for the mental health of survivors of violence in South Asia*, with approval for qualitative data collection from the University College London Research Ethics Committee (2744/007) and the Sangath Institutional Review Board (AN_2018_46). Clients gave signed consent for anonymised or pseudonymised use of their data at initial registration. They were assured of confidentiality, particularly that information would not be shared with their family, community, or the media. Counsellors informed clients of their right to access their records, including for use in building legal evidence.

REFERENCES

1. Hall BJ, Tucker JD. Surviving in place: the coronavirus domestic violence syndemic. *Asian J Psychiatry* 2020;53:102179.
2. Mazza M, Marano G, Lai C, et al. Danger in danger: Interpersonal violence during COVID-19 quarantine. *Psychiatry Res* 2020;289:113046. doi: 10.1016/j.psychres.2020.113046 [published Online First: 2020/11/27]

For peer review only

For peer review only - http://bmjopen.bmj.com/site/about/guidelines.xhtml

3. Evans ML, Lindauer M, Farrell ME. A Pandemic within a Pandemic - Intimate Partner Violence during Covid-19. *N Engl J Med* 2020;383(24):2302-04. doi: 10.1056/NEJMp2024046 [published Online First: 2020/09/17]
4. Bradbury-Jones C, Isham L. The pandemic paradox: The consequences of COVID-19 on domestic violence. *J Clin Nurs* 2020;29(13-14):2047-49. doi: 10.1111/jocn.15296 [published Online First: 2020/04/14]
5. UN Women. The shadow pandemic: violence against women during COVID-19 2021 [Available from: https://www.unwomen.org/en/news/in-focus/in-focus-gender-equality-in-covid-19-response/violence-against-women-during-covid-19 accessed 8 Feb 2021.
6. Viero A, Barbara G, Montisci M, et al. Violence against women in the Covid-19 pandemic: A review of the literature and a call for shared strategies to tackle health and social emergencies. *Forensic Sci Int* 2020;319:110650. doi: 10.1016/j.forsciint.2020.110650 [published Online First: 2020/12/20]
7. Sah LK, Sah P, Yadav MK, et al. Risk of gender-based violence and poor mental health among Nepalese women during COVID-19 pandemic: a review. *Nepal Med J* 2020;3(2):39-46.
8. UN Women. Impact of COVID-19 on violence against women and girls and service provision: UN Women rapid assessment and findings. COVID-19 Response. New York NY: United Nations Women, 2020.
9. Sánchez OR, Vale DB, Rodrigues L, et al. Violence against women during the COVID-19 pandemic: An integrative review. *Int J Gynaecol Obstet* 2020;151(2):180-87. doi: 10.1002/ijgo.13365 [published Online First: 2020/09/04]
10. Pfitzner N, Fitz-Gibbon K, True J. Responding to the 'shadow pandemic': practitioner views on the nature of and responses to violence against women in Victoria, Australia during the COVID-19 restrictions. Clayton VIC: Monash University, 2020.
11. Hsu LC, Henke A. COVID-19, staying at home, and domestic violence. *Rev Econ Househ* 2020;Nov 20:1-11.
12. Agüero JM. COVID-19 and the rise of intimate partner violence. *World Dev* 2021;137:105217. doi: 10.1016/j.worlddev.2020.105217 [published Online First: 2020/10/06]
13. Walklate S, Godfrey B, Richardson J. Changes and continuities in police responses to domestic abuse in England and Wales during the Covid-19 'lockdown'. *Policing and Society* 2021:1-13. doi: 10.1080/10439463.2021.1896514
14. Pinchoff J, Austrian K, Rajshekhar N, et al. Gendered economic, social and health effects of the COVID-19 pandemic and mitigation policies in Kenya: evidence from a prospective cohort survey in Nairobi informal settlements. *BMJ open* 2021;11(3):e042749. doi: 10.1136/bmjopen-2020-042749 [published Online First: 2021/03/05]
15. Sharma P, Khokhar A. Domestic violence and coping strategies among married adults during lockdown due to Coronavirus disease (COVID-19) pandemic in India: a cross-sectional study. *Disaster Med Public Health Prep* 2021:1-29. doi: 10.1017/dmp.2021.59 [published Online First: 2021/03/04]
16. Fraser E. Impact of COVID-19 pandemic on violence against women and girls. VAWG Helpdesk Research Report No 284. London: VAWG Helpdesk, 2020.
17. World Bank Group. Gender dimensions of the COVID-19 pandemic. Policy Notes. Washington DC: World Bank, 2020.
18. Majumdar S, Wood G. UNTF EVAW Briefing Note on the impact of COVID-19 on violence against women through the lens of Civil Society and Women's Rights Organizations. New York NY: UN Trust Fund to End Violence Against Women, 2020.
19. Gosangi B, Park H, Thomas R, et al. Exacerbation of Physical Intimate Partner Violence during COVID-19 Pandemic. *Radiology* 2021;298(1):E38-e45. doi: 10.1148/radiol.2020202866 [published Online First: 2020/08/14]

For peer review only - http://bmjopen.bmj.com/site/about/guidelines.xhtml

20. Evans DP. COVID-19 and violence: a research call to action. *BMC Womens Health* 2020;20(1):249. doi: 10.1186/s12905-020-01115-1 [published Online First: 2020/11/12]
21. WHO. COVID-19 and violence against women. What the health sector/system can do. Geneva: World Health Organization, 2020.
22. Vora M, Malathesh BC, Das S, et al. COVID-19 and domestic violence against women. *Asian J Psychiatr* 2020;53:102227-27. doi: 10.1016/j.ajp.2020.102227 [published Online First: 2020/06/15]
23. Krishnakumar A, Verma S. Understanding Domestic Violence in India During COVID-19: a Routine Activity Approach. *Asian J Criminol* 2021:1-17. doi: 10.1007/s11417-020-09340-1 [published Online First: 2021/03/17]
24. United Nations Human Settlements Programme (UN-Habitat). The challenge of slums: global report on human settlements 2003. London and Sterling VA: Earthscan Publications Ltd 2003.
25. Golechha M. COVID-19 Containment in Asia's Largest Urban Slum Dharavi-Mumbai, India: Lessons for Policymakers Globally. *J Urban Health* 2020;97(6):796-801. doi: 10.1007/s11524-020-00474-2 [published Online First: 2020/08/21]
26. Slater J, Masih N, Parth MN. In one of the world's largest slums, the fight against the coronavirus has turned into a struggle to survive. *Washington Post* 2020 12 May 2020.
27. Ray D, Subramanian S, Vandewalle L. India's lockdown. CEPR Policy Insight No 102. London: Centre for Economic Policy Research, 2020.
28. Mahapatro M, Prasad MM, Singh SP. Role of Social Support in Women facing Domestic Violence during Lockdown of Covid-19 while Cohabiting with the Abusers: Analysis of Cases Registered with the Family Counseling Centre, Alwar, India. *J Family Issues* 2021;January 2021:0192513X20984496. doi: 10.1177/0192513x20984496
29. Singh S, Bhattacharyya R. A review of domestic violence against women in India during lockdown. *Int J Innovation Creativity Change* 2020;14(6)
30. UN Women. COVID-19 and essential services provision for survivors of violence against women and girls. COVID-19 Response. New York NY: United Nations Women, 2020.
31. Bourgault S, Peterman A, O'Donnell M. Violence against women and children during COVID-19—one year on and 100 papers in. Washington DC: Center for Global Development, 2021.
32. Daruwalla N, Kanougiya S, Gupta A, et al. Prevalence of domestic violence against women in informal settlements in Mumbai, India: a cross-sectional survey. *BMJ open* 2020;10(12):e042444. doi: 10.1136/bmjopen-2020-042444 [published Online First: 2020/12/18]
33. Ritchie J, Spencer L. Qualitative data analysis for applied policy research. In: Bryman A, Burgess RG, eds. Analysing qualitative data. London and New York: Routledge 1994:173-94.
34. Gale NK, Heath G, Cameron E, et al. Using the framework method for the analysis of qualitative data in multi-disciplinary health research. *BMC Med Res Methodol* 2013;13:117. doi: 10.1186/1471-2288-13-117 [published Online First: 2013/09/21]
35. Srivastava A, Thomson SB. Framework Analysis: a qualitative methodology for applied policy research. *Journal of Administration & Governance* 2009;4(2):72-79.
36. Attride-Stirling J. Thematic networks: an analytic tool for qualitative research. *Qualitative Research* 2001;1(3):385-405. doi: 10.1177/146879410100100307
37. Lacey A, Luff D. Qualitative data analysis. Yorkshire & the Humber: National Institute for Health Research 2007.
38. Pope C, Ziebland S, Mays N. Qualitative research in health care: analysing qualitative data. *British Medical Journal* 2000;320:114-16.
39. Smith J, Firth J. Qualitative data analysis: the framework approach. *Nurse Researcher* 2011;18:52-62.
40. Renzetti CM, Campe M. Feminist praxis and gender violence. In: Naples NA, ed. Companion to feminist studies. Chichester: Wiley-Blackwell 2020:411-26.

For peer review only

41. Nigam S. COVID-19: India's response to domestic violence needs rethinking. *South Asia J* 2020;Available at SSRN: https://ssrn.com/abstract=3598999
42. Hamadani JD, Hasan MI, Baldi AJ, et al. Immediate impact of stay-at-home orders to control COVID-19 transmission on socioeconomic conditions, food insecurity, mental health, and intimate partner violence in Bangladeshi women and their families: an interrupted time series. *The Lancet Global health* 2020;8(11):e1380-e89. doi: 10.1016/s2214-109x(20)30366-1 [published Online First: 2020/08/29]
43. Al-Ali N. Covid-19 and feminism in the Global South: Challenges, initiatives and dilemmas. *European Journal of Women's Studies* 2020;27(4):333-47. doi: 10.1177/1350506820943617
44. Moreira DN, Pinto da Costa M. The impact of the Covid-19 pandemic in the precipitation of intimate partner violence. *Int J Law Psychiatry* 2020;71:101606. doi: 10.1016/j.ijlp.2020.101606 [published Online First: 2020/08/10]
45. Jarnecke AM, Flanagan JC. Staying safe during COVID-19: How a pandemic can escalate risk for intimate partner violence and what can be done to provide individuals with resources and support. *Psychol Trauma* 2020;12(S1):S202-s04. doi: 10.1037/tra0000688 [published Online First: 2020/06/20]
46. Akel M, Berro J, Rahme C, et al. Violence Against Women During COVID-19 Pandemic. *Journal of Interpersonal Violence* 2021:0886260521997953. doi: 10.1177/0886260521997953
47. Ragavan MI, Garcia R, Berger RP, et al. Supporting Intimate Partner Violence Survivors and Their Children During the COVID-19 Pandemic. *Pediatrics* 2020;146(3) doi: 10.1542/peds.2020-1276 [published Online First: 2020/08/21]
48. Felson M, Cohen LE. Human ecology and crime: a routine activity approach. *Human Ecology* 1980;8:389-406.
49. Campbell AM. An increasing risk of family violence during the Covid-19 pandemic: strengthening community collaborations to save lives. *Forensic Sci Int* 2020;Reports 2:100089.
50. Emezue C. Digital or digitally delivered responses to domestic and intimate partner violence during COVID-19. *JMIR Public Health Surveill* 2020;6:e19831.
51. UN Women. Prevention: violence against women and girls & COVID-19. COVID-19 Response. New York NY: United Nations Women, 2020.
52. Oxfam India. On women's backs: India inequality report 2020. New Delhi: Oxfam India, 2020.
53. Malik S, Naeem K. Impact of COVID-19 pandemic on women: health, livelihoods & domestic violence. Islamabad: Sustainable Development Policy Institute, 2020.
54. Agarwal B. Reflections on the Less Visible and Less Measured: Gender and COVID-19 in India. *Gender & Society* 2021;35(2):244-55. doi: 10.1177/08912432211001299
55. George J, Stith SM. An updated feminist view of intimate partner violence. *Fam Process* 2014;53(2):179-93. doi: 10.1111/famp.12073 [published Online First: 2014/04/23]
56. UN Women. From insights to action: gender equality in the wake of COVID-19. COVID-19 Response. New York NY: United Nations Women, 2020.
57. True J. The political economy of violence against women. Oxford: Oxford University Press 2012.
58. Maji S, Bansod S, Singh T. Domestic violence during COVID-19 pandemic: The case for Indian women. *J Community Appl Soc Psychol* 2021 doi: 10.1002/casp.2501 [published Online First: 2021/04/07]
59. Roy AS, Sen N, Bagchi SS. Gender-based violence in India in Covid-19 lockdown. *J Comparative Lit Aesthetics* 2021;44:41-55.
60. Schokkenbroek JM, Anrijs S, Ponnet K, et al. Locked Down Together: Determinants of Verbal Partner Violence During the COVID-19 Pandemic. *Violence Gender* 2021(5 Apr 2021) doi: 10.1089/vio.2020.0064
61. Béland L-P, Brodeur A, Haddad J, et al. COVID-19, family stress and domestic violence: remote work, isolation and bargaining power. IZA DP No 13332. Bonn: Institute of Labor Economics, 2020.

For peer review only - http://bmjopen.bmj.com/site/about/guidelines.xhtml

62. Chakraborty P. Distribution of fresh fruits and vegetables in Dharavi, India: lessons and learnings from the Covid-19 pandemic. Mumbai: Society for Nutrition, Education and Health Action, 2020.
63. Natarajan J. Women's safety during lockdown. *The Hindu* 2020 13 April 2020.
64. Peterman A, Potts A, O'Donnell M, et al. Pandemics and violence against women and children. Working Paper 528. Washington DC: Center for Global Development, 2020.

For peer review only

For peer review only - http://bmjopen.bmj.com/site/about/guidelines.xhtml

COREQ (COnsolidated criteria for REporting Qualitative research) Checklist

A checklist of items that should be included in reports of qualitative research. You must report the page number in your manuscript where you consider each of the items listed in this checklist. If you have not included this information, either revise your manuscript accordingly before submitting or note N/A.

Topic	Item No.	Guide Questions/Description	Reported on Page No.
**Domain 1: Research team and reflexivity**			
Personal characteristics			
Interviewer/facilitator	1	Which author/s conducted the interview or focus group?	5
Credentials	2	What were the researcher's credentials? E.g. PhD, MD	5
Occupation	3	What was their occupation at the time of the study?	5
Gender	4	Was the researcher male or female?	5
Experience and training	5	What experience or training did the researcher have?	5
Relationship with participants			
Relationship established	6	Was a relationship established prior to study commencement?	5
Participant knowledge of the interviewer	7	What did the participants know about the researcher? e.g. personal goals, reasons for doing the research	5
Interviewer characteristics	8	What characteristics were reported about the inter viewer/facilitator? e.g. Bias, assumptions, reasons and interests in the research topic	5
**Domain 2: Study design**			
Theoretical framework			
Methodological orientation and Theory	9	What methodological orientation was stated to underpin the study? e.g. grounded theory, discourse analysis, ethnography, phenomenology, content analysis	5
Participant selection			
Sampling	10	How were participants selected? e.g. purposive, convenience, consecutive, snowball	5
Method of approach	11	How were participants approached? e.g. face-to-face, telephone, mail, email	5
Sample size	12	How many participants were in the study?	6
Non-participation	13	How many people refused to participate or dropped out? Reasons?	6
Setting			
Setting of data collection	14	Where was the data collected? e.g. home, clinic, workplace	5
Presence of non-participants	15	Was anyone else present besides the participants and researchers?	5
Description of sample	16	What are the important characteristics of the sample? e.g. demographic data, date	6
Data collection			
Interview guide	17	Were questions, prompts, guides provided by the authors? Was it pilot tested?	5
Repeat interviews	18	Were repeat inter views carried out? If yes, how many?	0
Audio/visual recording	19	Did the research use audio or visual recording to collect the data?	0
Field notes	20	Were field notes made during and/or after the inter view or focus group?	5
Duration	21	What was the duration of the inter views or focus group?	5
Data saturation	22	Was data saturation discussed?	0
Transcripts returned	23	Were transcripts returned to participants for comment and/or	0

For peer review only

Topic	Item No.	Guide Questions/Description	Reported on Page No.
		correction?	
**Domain 3: analysis and findings**			
Data analysis			
Number of data coders	24	How many data coders coded the data?	5
Description of the coding tree	25	Did authors provide a description of the coding tree?	5
Derivation of themes	26	Were themes identified in advance or derived from the data?	5
Software	27	What software, if applicable, was used to manage the data?	5
Participant checking	28	Did participants provide feedback on the findings?	0
Reporting			
Quotations presented	29	Were participant quotations presented to illustrate the themes/findings? Was each quotation identified? e.g. participant number	6-8
Data and findings consistent	30	Was there consistency between the data presented and the findings?	6-8
Clarity of major themes	31	Were major themes clearly presented in the findings?	6-8
Clarity of minor themes	32	Is there a description of diverse cases or discussion of minor themes?	9

Developed from: Tong A, Sainsbury P, Craig J. Consolidated criteria for reporting qualitative research (COREQ): a 32-item checklist for interviews and focus groups. *International Journal for Quality in Health Care*. 2007. Volume 19, Number 6: pp. 349 – 357

Once you have completed this checklist, please save a copy and upload it as part of your submission. DO NOT include this checklist as part of the main manuscript document. It must be uploaded as a separate file.

For peer review only

BMJ Open

Intersectional tension: a qualitative study of the effects of the Covid-19 response on survivors of violence against women in urban India

Journal:	*BMJ Open*
Manuscript ID	bmjopen-2021-050381.R2
Article Type:	Original research
Date Submitted by the Author:	08-Sep-2021
Complete List of Authors:	Huq, Mita; UCL, Institute for Global Health<br>Das, Tanushree; Society for Nutrition, Education & Health Action, Program on Prevention of Violence Against Women and Children<br>Devakumar, Delan; University College London, Institute for Global Health<br>Daruwalla, Nayreen; Society for Nutrition, Education & Health Action, Program on Prevention of Violence Against Women and Children<br>Osrin, David; University College London, Institute for Global Health
**Primary Subject Heading**:	Public health
Secondary Subject Heading:	Global health
Keywords:	COVID-19, Public health < INFECTIOUS DISEASES, MENTAL HEALTH, QUALITATIVE RESEARCH

SCHOLARONE™ Manuscripts

I, the Submitting Author has the right to grant and does grant on behalf of all authors of the Work (as defined in the below author licence), an exclusive licence and/or a non-exclusive licence for contributions from authors who are: i) UK Crown employees; ii) where BMJ has agreed a CC-BY licence shall apply, and/or iii) in accordance with the terms applicable for US Federal Government officers or employees acting as part of their official duties; on a worldwide, perpetual, irrevocable, royalty-free basis to BMJ Publishing Group Ltd ("BMJ") its licensees and where the relevant Journal is co-owned by BMJ to the co-owners of the Journal, to publish the Work in this journal and any other BMJ products and to exploit all rights, as set out in our licence.

The Submitting Author accepts and understands that any supply made under these terms is made by BMJ to the Submitting Author unless you are acting as an employee on behalf of your employer or a postgraduate student of an affiliated institution which is paying any applicable article publishing charge ("APC") for Open Access articles. Where the Submitting Author wishes to make the Work available on an Open Access basis (and intends to pay the relevant APC), the terms of reuse of such Open Access shall be governed by a Creative Commons licence – details of these licences and which Creative Commons licence will apply to this Work are set out in our licence referred to above.

Other than as permitted in any relevant BMJ Author's Self Archiving Policies, I confirm this Work has not been accepted for publication elsewhere, is not being considered for publication elsewhere and does not duplicate material already published. I confirm all authors consent to publication of this Work and authorise the granting of this licence.

For peer review only

Intersectional tension: a qualitative study of the effects of the Covid-19 response on survivors of violence against women in urban India

AUTHORS

Mita Huq

Institute for Global Health, University College London, London, WC1N IEH, UK

mita.huq.19@ucl.ac.uk

Tanushree Das

Program on Prevention of Violence Against Women and Children, SNEHA, Mumbai, Maharashtra, 400017, India

tanushree.das@snehamumbai.org

Delan Devakumar

Institute for Global Health, University College London, London, WC1N IEH, UK

d.devakumar@ucl.ac.uk

Nayreen Daruwalla

Program on Prevention of Violence Against Women and Children, SNEHA, Mumbai, Maharashtra, 400017, India

nayreen@snehamumbai.org

David Osrin (**corresponding author)**

Institute for Global Health, University College London, London, WC1N IEH, UK

d.osrin@ucl.ac.uk

For peer review only

For peer review only - http://bmjopen.bmj.com/site/about/guidelines.xhtml

ABSTRACT

Objectives

There is concern worldwide that efforts to address the SARS-CoV-2 pandemic have affected the frequency and intensity of domestic violence against women. Residents of urban informal settlements faced particularly stringent conditions during the response in India. Counsellors spoke with registered survivors of domestic violence in Mumbai, with two objectives: to understand how the pandemic and subsequent lockdown had changed their needs and experiences, and to recommend programmatic responses.

Design

Qualitative interviews and framework analysis.

Setting

A non-government support program for survivors of violence against women, providing services mainly for residents of informal settlements.

Participants

During follow-up telephone counselling with survivors of violence against women who had previously registered for support and consented to the use of information in research, counsellors took verbal consent for additional questions about the effects of Covid-19 on their daily life, their ability to speak with someone, and their counselling preferences. Responses were recorded as written notes.

Results

The major concerns of 586 clients interviewed between April and July 2020 were meeting basic needs (financial stress, interrupted livelihoods, and food insecurity), confinement in small homes (family tensions and isolation with abusers), and limited mobility (power imbalances in the home and lack of opportunity for disclosure and stress relief). A major source of stress was the increased burden of unpaid domestic care, which fell largely on women.

Conclusion

The Covid-19 pandemic has increased the burden of poverty and gendered unpaid care. Finance and food security are critical considerations for future response, which should consider inequality, financial support, prioritising continued availability of services for survivors of violence, and expanding access to social networks. Decision-makers must be aware of the gendered, intersectional effects of interventions and must include residents of informal settlements who are survivors of domestic violence in the planning and implementation of public health strategies.

KEYWORDS

Covid-19; Domestic Violence; Gender-based violence; poverty areas; Mumbai; India

For peer review only

Article summary

Strengths and limitations of this study

Strengths

- Interviews were conducted with almost 600 survivors of violence.
- Existing relationships between survivors and counsellors.
- All participants were residents of urban informal settlements in which stringent measures to limit the spread of Covid-19 were implemented.

Limitations

- The study did not examine incidence of domestic violence against women who were not already registered.
- Telephone interviews were limited by women's willingness to provide information.

For peer review only

For peer review only - http://bmjopen.bmj.com/site/about/guidelines.xhtml

BACKGROUND

As governments implemented measures to address the spread of SARS-CoV-2, organisations working to prevent violence against women became concerned that the measures might themselves give rise to or escalate domestic violence.[1-5] This concern was informed by three strands of evidence. The first was that states and civil society organisations noted large increases in contacts through domestic violence helplines and websites at the same time as the numbers of face-to-face consultations fell.[6-13] The second was that the conditions imposed by the pandemic, such as lockdown and income loss, were likely to increase household and interpersonal conflict.[14 15] The third was that public health emergencies have been shown repeatedly to be followed by increases in violence against women and girls.[16 17] Amid reports that the frequency and severity of domestic violence were increasing,[17-20] the World Health Organization (WHO) and United Nations Women (UN Women) raised concerns that measures such as quarantine and lockdown could increase the risk of violence against women.[8 21]

These concerns were observable in India, where an increase in violence against women was recognised in the media and some studies.[22 23] The reports describe a relationship between loss of economic prospects, separation from friends and family, and amplified gendered expectations as a result of lockdown measures. Lockdown measures were implemented suddenly and stringently, particularly in urban informal settlements (slums). Residents of such settlements, in which ~40% of Mumbai's households are located, were seen as hotspots for contagion because of density and overcrowding, insubstantial housing, and insufficient water and sanitation.[24 25] An average of five people share one or two rooms, most rely on shared toilets, and around two-thirds need to leave the house to get water. The government of Maharashtra announced a lockdown in Mumbai on 23rd March 2020, for an initial 21 days with subsequent extensions. Major informal settlements were quarantined as red zones,[26] their access barricaded, public toilets disinfected, and a police-enforced curfew instituted. Residents were confined to their homes unless they faced medical emergencies, transport services and vehicular movement curtailed, and industry and retail outlets closed. Residents were screened for SARS-CoV-2 through door-to-door visits by public and private sector healthcare workers and centres providing free food and healthcare were set up in schools and halls.[25]

These measures were commended for preventing a surge in cases in areas where the potential spread of SARS-CoV-2 was particularly concerning,[25 27] but fear of institutional quarantine was common and accompanied by a degree of community stigma. The risk of domestic violence does not appear to have been considered. At the same time as survivors of violence were potentially confined with their abusers, organisations working to prevent and respond to violence suffered resource limitations,[28 29] reduced capacities, and interrupted monitoring and evaluation activities.[8 21 30] The civil society work of SNEHA (Society for Nutrition, Education and Health Action) illustrates these pressures. Since 2000, the SNEHA Program on Prevention of Violence Against Women and Children has provided support to over 13 000 survivors of violence against women. Services are open to all—particularly residents of Mumbai's informal settlements—and are delivered from five community and three public hospital counselling centres. Support is available through a combination of in-house services and liaison. In-house human resources include community outreach workers, counsellors, legal advisors and lawyers, and psychologists. These are complemented by liaison with hospital practitioners, the police, District Legal Aid services, shelters, and psychiatrists.

As the response to the pandemic unfolded, the programme moved counselling and crisis intervention services online. Existing helplines were augmented and survivors of violence could call or email counselling centres for assistance. A cadre of 300 volunteers gave out contact details in communities. At the same time, counsellors contacted their existing clients and offered to provide follow-up telephonically. SNEHA developed a protocol for this follow-up, based on existing safety protocols and emphasising confidentiality and the same client-

For peer review only - http://bmjopen.bmj.com/site/about/guidelines.xhtml

centred approach followed in face-to-face sessions. It became clear that these telephone conversations could provide a rich understanding of women's experiences of domestic violence in urban informal settlements during lockdown. In discussion with counsellors, we designed a topic guide for qualitative interviews that they could integrate into their discussion with existing clients. Our objectives were to use the interviews to understand how the Covid-19 pandemic and subsequent lockdown changed women's needs and experiences, and to recommend programmatic responses to domestic violence in light of pandemics and the strategies used to mitigate them.

METHODS

Setting and participants

Our study explored the experiences of survivors of domestic violence in a specific context:[31] residents of urban informal settlements in Mumbai whose suffering as a consequence of domestic violence by either an intimate partner or another family member had led them to register for support. Only about 8% of women in these circumstances get as far as registering with a support organisation,[32] and the sample therefore represents a selected group. Interviewees were women clients who had been interacting with SNEHA counsellors prior to the lockdown and had developed relationships with them. They were all survivors of violence, having connected with the organisation after surviving domestic violence—physical, sexual, emotional, or economic violence, coercive control or neglect—by intimate partners or family members. Since they were all survivors, our interest was in changes in their experience rather than in the incidence of violence against women who had not previously made contact.

Data collection

Eleven female counsellors with postgraduate education in social work or psychology and between two and ten years of experience of supporting clients from our centres conducted interviews after receiving project-specific training. Clients had registered initially through self-report, referral from agencies such as the police, clinicians, or non-government organizations, or referral by SNEHA community organisers or volunteers trained to identify and engage with survivors. Participants were contacted by their counsellors for telephone follow-up between March and May 2020. Counsellors explained that, as part of their conversations, they were doing a study to understand women's experiences during the lockdown. With agreement from clients, they administered a semi-structured interview about how Covid-19 had affected their daily life, family life, ability to speak with someone, and their counselling preferences. Interviews were conducted during working hours. If a client did not answer the phone, nearby field staff, volunteers, or women's group members would visit her and encourage her to contact her counsellor to indicate her availability. Counsellors provided all clients with information on Covid-19 (transmission, social distancing, handwashing, masks, emotional hygiene, anxiety management, freedom to contact the organisation for advice). If a family member answered the phone (or it seemed likely to the counsellor that someone was listening in), they said that the call was part of the Covid-19 prevention program and that they were interested in the family's emotional health. Counsellors recorded responses in field notes categorised according to the topic guide (Supplementary file). Interviews were conducted in Hindi and Marathi and subsequently summarised in English by bilingual team leaders. Interviews were part of counselling calls that lasted up to an hour. All potential identifiers were removed from the dataset.

Data analysis

The familiarisation process began by reading all the records. Two authors approached the data independently (MH and DO). We used the framework approach to thematic analysis.[33] Increasingly used in health research,[34] this approach lends itself to studies by multidisciplinary teams with fairly specific objectives and questions, limited time for data collection, and a predefined large sample.[35] Data are charted in a matrix in which rows

For peer review only - http://bmjopen.bmj.com/site/about/guidelines.xhtml

represent interviewees and columns represent codes, and information can be compared across cases and codes. We identified some of these codes before the analysis and augmented them with codes that emerged.[33 36-39] The two authors who analysed the data compared their codes iteratively. The emergent themes were discussed over four iterations, with inputs from all authors. An indexing and data exploration process expanded this framework by inductively adding themes and sub-themes.

Patient and public involvement

Clients of our counselling centres for survivors of violence against women were first involved during telephonic discussions with counsellors. The research questions were a direct response to concerns about their safety and emotional health and wellbeing during the response to the Covid-19 pandemic, and sought to understand their experiences and preferences for services. Dissemination will involve personal feedback of the findings to clients during subsequent counselling sessions and feedback during regular community mobilisation meetings in informal settlements across the city.

RESULTS

Participants

Between 1st April and 30th July 2020, counsellors spoke with 720 clients. Our analysis draws on interviews with 586 women for whom complete background information was available. At the time of interview, 54% of respondents were in the first month of lockdown, 40% in the second, and 6% in the third month or later. Of the existing clients who provided information, 46% had been seeing their counsellor for less than six months, 33% for up to a year, 12% for up to 18 months, and 8% for longer periods. Table 1 shows that most women were in their 20s or 30s (80%) and had attended school to at least secondary level (74%). Most had been married at some point (92%) and around two-thirds had children (63%). The largest group had been living in nuclear families at the time of initial consultation (59%) and the next largest group in joint families with their in-laws (33%). Most lived in informal settlements (*zopadpatti*) or former workers' accommodation (*chawls*) (73%). The major religions were about equally represented. Around one-third of women (32%) worked outside the home, usually in the informal sector.

The background to initial consultation for most women had been intimate partner violence (70%). However, 44% had suffered violence by another family member and in 28% of cases violence came from both intimate partners and other family members. Family violence was predominantly emotional (87%), economic (63%), and physical (54%), and coercive control (46%). The primary concerns that women said had led them to consult were economic abuse, particularly feeling that they had been denied money (56%), physical violence (49%), being compelled to leave their home (32%), being threatened by their partner (25%), having had their property or possessions taken by family members (19%), and suffering sexual violence (17%). Although instances of violence were not always the triggers for consultation, 86% of women described experiences of emotional violence, 76% economic violence, 64% physical violence, 56% neglect, 56% controlling behaviours, and 35% sexual violence. Most had survived more than one of these forms of violence (86%) and 22% all six forms.

For peer review only - http://bmjopen.bmj.com/site/about/guidelines.xhtml

Table 1. Participant characteristics at registration for counselling

**Age (years)**	**n**	**(%)**
Under 20	26	(4)
20-29	262	(45)
30-39	205	(35)
40-49	56	(10)
50+	37	(6)
**Education**		
None	63	(11)
Primary	78	(13)
Lower secondary	97	(16)
Higher secondary	226	(39)
Higher	110	(19)
Unknown	12	(2)
**Marital status**		
Married	431	(73)
De facto	16	(3)
Separated or divorced	64	(11)
Widowed	29	(5)
Unmarried	45	(8)
**Children**		
None	217	(37)
1	149	(25)
2 or more	220	(38)
**Family arrangement**		
Nuclear	342	(59)
Joint	194	(33)
Extended	23	(4)
Living alone	19	(3)
Other	8	(1)
**Religion**		
Hindu	278	(47)
Muslim	241	(41)
Other	67	(12)
**Remunerated employment**		
None	392	(67)
Student	7	(1)
Informal sector	127	(22)
Formal sector	60	(10)
All	586	(100)

A matrix of stressors

What emerged from women's accounts of life during lockdown was a matrix of stressors that interacted with domestic violence: worry about basic needs, confinement in small homes, limited mobility, concern for children, and alcohol. Lockdown had confined them to four general living arrangements: a nuclear family, a joint family with husband and in-laws, with members of a natal family, or either alone or with children. At the time of interview, around half of respondents (52%) were living with their partner in either nuclear or joint families. Most women who were not had relocated to live with their natal family. This usually represented a longer-term strategy of separation, but for some a short visit had become longer as a result of lockdown. For women who were confined in a nuclear family with a husband who had been abusive at the time of initial consultation, 27% were still surviving

For peer review only

violence. For women who were living with a husband and in-laws, 28% were still surviving intimate partner or family violence.

Worry about basic needs

Dominant in women's narratives were problems meeting basic needs. Half of respondents expressed overwhelming concerns about money and food, and most women who said that they were managing were drawing on their own or their family's savings. Families living in informal settlements depend largely on a mosaic of livelihoods in the informal sector and most women said that the lockdown had caused work to be suspended. Restrictions on movement meant that daily earners could not go to work: "I used to do domestic work but am now unable to go to work. My employers have been calling me to say that because … Covid-19 cases are high here, they don't want me to join work for another six months. I'm worried about finances" (20-29 years, separated with 1 child and living with natal family; physical, emotional, and economic IPV at first consultation).

A few days of lockdown led many families to draw on their limited savings. "We're running out of savings. My husband's boss is not able to pay him right now but is providing him with the basics so he's staying at the job. But my kids and I are struggling to make ends meet" (30-39 years, married with 4 children and living with husband in nuclear family; physical, emotional, economic IPV). The only safety net was the government ration system, itself compromised by halts in supply and quarantine of communities. Families who were eligible for rations became more dependent on them, but the quantity and quality of rations and food in the shops was compromised by supply problems. "The food resources have been insufficient, as well as the whole family is present and two meals for all become a little tough with this current crisis" (20-29 years, unmarried and living in natal family; physical IPV). Food insecurity therefore resulted from both lack of money and unavailability of food: "All shops are closed. The area is sealed. We are managing with the rations we get from the ration shop. Other shops are closed. Around 15 days back a lady had come to ask for our contact numbers to provide help, but we haven't heard from her or anyone so far" (20-29 years, unmarried and living in natal family; physical family violence). Coupled with inconsistent or insufficient government and non-government assistance, these pressures made members of food insecure families leave the home in contravention of lockdown policy to seek food, exposing themselves to SARS-CoV-2 and punitive action by law enforcement. The combination of insecurity and violence was a potent stressor: "Barely being able to manage. My husband came and fought with me a lot a couple of days ago. After that now the shop below my house is giving me stuff on loan" (20-29 years, married with 3 children and living with children; physical, sexual, emotional, economic IPV).

Confinement in small homes

Stress was increased by the small living spaces in informal settlements in which most respondents lived. Lack of privacy and time alone exacerbated tension in large families, whereas loneliness was reported by women who lived alone or were being forcibly isolated or neglected. Tension was often increased by a combination of overcrowding and precarity. "… I'm in tension and stress due to lack of money and resources. Sometimes we fight with each other, but … I'm not able to go outside…" (40-49 years, married with 6 children and living with husband in nuclear family; physical, sexual, emotional, economic IPV). Women living in joint families faced predominantly emotional violence, and physical violence in three cases.

Proximity could, however, lead to improvements. For women living with abusive husbands, previously acrimonious relations sometimes improved: "… My husband came closer to me and my daughter, taking care of both of us" (30-39 years, married with 1 child and living with husband in nuclear family; physical, emotional, economic IPV). It was also possible for violence to decrease because of wariness rather than benevolence, when the abuser relied on the respondent: "My husband fights with me all the time. He constantly bickers. He's not beating me right now because he knows he can't do without me for now" (30-39 years,

For peer review only - http://bmjopen.bmj.com/site/about/guidelines.xhtml

married with 3 children and living with husband in joint family; physical, sexual, emotional, economic IPV and family violence).

Nevertheless, most women living with their natal family—away from the source of violence—were positive about it: “I love taking care of my family and being with them. Glad they support me so much. We're very happy we're getting quality time together” (30-39 years, separated with no children and living with natal family; physical, sexual, emotional, economic IPV).

Limited mobility

The effects of precarity and confinement were exacerbated by the limits set to mobility. These had two general effects: manifestation of power imbalances and an inability to find relief outside the home. For some women, confinement had “… given my husband more power. It's very difficult for us” (30-39 years, married with 4 children and living with husband in nuclear family; physical, sexual, emotional, economic IPV). Money was withheld or taken from women and abusers controlled their movements indoors, forced them out of the house, or isolated them socially. “My husband and in-laws are very abusive. Many times they have locked me up and left me without food” (20-29 years, married with no children and living with husband in joint family; physical, sexual, emotional, economic IPV and family violence). A few survivors said that their partners had not allowed them to access medical care on the pretext of acquiring infection.

Confinement also led to feelings of helplessness and lack of opportunity to decompress. Of the 208 women who had not reported physical violence at first consultation, four said that it had begun during the lockdown: “My husband beat me for the first time… My husband is getting violent and I'm alone at home and neither of us can leave to cool down. I feel trapped” (20-29 years, married with no children and living with husband in nuclear family; emotional IPV and family violence). Threats of violence from their abusers prevented women from disclosing their unhappiness. This was particularly concerning for respondents in abusive situations in need of counselling services, where the abuser was always around or monitoring their calls. Counsellors sometimes noted reticence in women’s answers to questions: “Client's husband was there and so she was being a little calculating about what she was saying” (20-29 years, married with 3 children and living with husband in nuclear family; physical, sexual, emotional, economic IPV). Women were unable to draw on emotional support outside the home from neighbours, friends, or family members. “Before this situation, I spent some time with my neighbours to share my feelings and thoughts, but now due to more housework, I’m not able to manage more time for myself” (30-39 years, married with 4 children and living with husband in nuclear family; physical, emotional, economic IPV and family violence). Women described hesitancy to disclose their emotions to others for fear of being judged or misunderstood, or because they had limited access to phones. They often felt that it was their responsibility to handle their mental health since the pandemic had already brought enough stress and fear to those around them. “I don't share with anyone. I don't want to trouble them. I don't talk to my family and I don't have any friends here. So I keep everything to me” (20-29 years, separated with 1 child and living with natal family; physical, sexual, emotional, economic IPV and family violence).

Concern for children

Beside their concern for their children’s diet, education, and socialisation, women were worried that fights, harassment, or physical violence that would otherwise have occurred while children were at school or outside the home now happened in front of them. One woman’s husband was “…staying at home the whole day … and that has been affecting the mental health of my kids also as they are afraid of the rage that they see after their father is drunk and acts aggressive or hits me” (30-39 years, married with 3 children and living with husband in nuclear family; physical, sexual, emotional, economic IPV).

Alcohol

For peer review only - http://bmjopen.bmj.com/site/about/guidelines.xhtml

Lockdown changed the availability of alcohol, leading to improvements in some households, but more drinking at home or withdrawal symptoms in others. “He has been creating a lot of issues being drunk all day and also forces me to have sex sometimes” (30-39 years, living with partner with 2 children in nuclear family; physical, sexual, emotional, economic IPV). For those reporting improvement, it was primarily through less drinking and more time spent caring for the family. “My husband has stopped drinking so there is some peace at home” (20-29 years, married with 3 children in nuclear family; physical, sexual, emotional, economic IPV). In some homes where drinking persisted, not only were tensions heightened, but abusers would leave the house to find alcohol. This caused further worries about exposure to SARS-CoV-2 and added to household fears for health and wellness.

“Women’s work”

Along with their concerns about basic needs, many women said that their main source of stress was the increase in household responsibilities that fell disproportionately on them. Some of these related to hygiene: cleaning, disinfection, and childcare in an environment in which viral transmission was a major concern. The extra housework occasioned by more people in the household and children being off school was usually shouldered by women. “I have extra workload and no help, while if I expect help my husband and mother-in-law abuse me” (30-39 years, married with 2 children and living with husband in joint family; physical, emotional, economic IPV). Women tended to be tasked with cooking and cleaning and men were tasked with leaving the house to get supplies. The cognitive effects of these responsibilities centred on weariness and frustration with gender norms, when families were “… using the lockdown for harassment and intimidation” (20-29 years, married with no children and living with husband in joint family; physical, sexual emotional, economic IPV and family violence).

Respondents who also worked from home reported difficulty balancing employment with their caring responsibilities, saying that family members had heightened expectations that they would complete chores. “I have to work more than before and my sister-in-law keeps saying that if I don’t work I am just another baggage using their resources” (20-29 years, married with no children and living with husband in joint family; physical, emotional, economic IPV). For these households, domestic labour ranked higher than work and school, and violating expectations could trigger abuse: “My natal family does emotional violence on me because I work from home, so the full day I am spending time at home, my family expects me to do domestic work” (20-29 years, divorced with 1 child and in joint family; emotional family violence). Even respondents who had moved in with their natal family said that they felt guilty for burdening them as they were unable to contribute financially, and that their presence added to precarity. “I am staying with my natal family and now that I am unable to contribute … I feel awkward and a burden to them. Not that they ever verbalized it, but the non-verbal gestures are enough to understand” (40-49 years, married with no children and living in natal family; physical, emotional, economic family violence).

Addressing needs with survivor-centred approaches

Respondents highlighted several needs during their interviews, many of them being basic survival supplies such as rations and money. Others flagged the need for legal counsel and continued court proceedings to address their domestic violence cases, all of which were interrupted by lockdown. A few needed medical attention and some simply wanted to see their friends and families or take a walk as a moment of solace.

They also discussed their experience with tele-counselling. Since all the respondents were survivors of violence with prior connections to counsellors, we asked how tele-counselling fit with their circumstances before and during the pandemic. Respondents recognised the importance of distanced counselling services during the pandemic, and generally found telephone discussion a good option. Some actually preferred it to in-person meetings, saying that it overcame geographic and physical barriers and time constraints that had prevented them from visiting a centre. Nevertheless, most said that they would prefer to return to the

For peer review only - http://bmjopen.bmj.com/site/about/guidelines.xhtml

counselling centre when it was safe. Respondents who reported substantial changes to their daily life said that they were unable to talk at home because of lack of privacy, family members listening in, higher risk of violence if discovered, or limited access to a phone. They said that in-person counselling was better because they felt more comfortable and articulate at the counselling centre and the services would be better. Respondents who did not report increased violence agreed with this, but needed different types of services from the centre, such as psychological counselling for family members other than themselves or legal guidance.

DISCUSSION

During the response to the Covid-19 pandemic, survivors of domestic violence in Mumbai's urban informal settlements faced—as well as fears of contagion—heightened financial and food insecurity, confinement in small homes in the presence or absence of abusers, coercive control, inability to discuss their experiences with others, and an increased burden of domestic labour. Legal processes were to all intents and purposes halted during the pandemic,[8 18] increasing the stress on clients who were waiting for resolution of their cases. Our study explored the needs and priorities of women whose relationships were already marked by intimate partner and family violence, and whose living conditions were already difficult. Despite seeking assistance from our counsellors, they lived in a state of constant fear about finances and food, an increased burden of household responsibilities, and tolerance of coercive behaviours.

Intersectional insecurity

Quarantine measures and restrictions on movement increased financial and food insecurity, linking to pathways of risk via economic constraint and exploitative relationships. All the respondents were already receiving support from our program. Although unacceptable, domestic violence was an existing feature of their lives on which the effects of lockdown played out. For some, confinement with their partner had led to an increase in physical violence, but their abiding concern was with finances and food security,[22] driven predominantly by loss of employment for either themselves or their family. The matrix of stressors echoes the concerns of intersectional feminism, which encourages broader consideration of social factors that shape survivorship.[40] The increased risk of violence in conditions in which the hardships associated with poverty and informal settlements are augmented is evident in India,[41] rural Bangladesh,[42] and in other lower-income countries.[43] We need to pay attention to survivors' basic needs: while some may benefit from exit strategies and social and legal protective services, there is a clear need for food and financial support.[14] Lack of availability of alcohol (or an increase in drinking at home)[9] led in some cases to increased violence by habitual users. In others, it seems to have diminished irritability and allowed users to exhibit impulse control.[28 29]

Confinement

Insecurity combines with enforced proximity with perpetrators of violence,[14 20 22 23 44 45] in small homes in urban informal settlements, amplifying the tension between family members and instances of domestic violence.[46] The global discussion tends to equate domestic violence with intimate partner violence, but violence by other family members is common in India. We have reported previously that other family members are responsible for around half of emotional violence against women in informal settlements in Mumbai.[32] Lockdown may confine a woman with her husband, but it may equally confine her with her marital family, including members of the extended family who are living together for the duration. This is exacerbated when perpetrators exploit the restrictions associated with the pandemic to exercise power and control,[6 47] including exploiting the fear of contagion.[4 9] Changes in household composition as a result of lockdown altered the situation in which violence occurred. This is an example of how social structure allows (or does not allow) people to translate criminal intentions into action. Routine activity theory suggests that an offence requires an offender, a suitable target, and absence of capable guardians, and it is

For peer review only - http://bmjopen.bmj.com/site/about/guidelines.xhtml

interesting to think about the effects of changes in who is present in the home as changes in a micro-ecology in which domestic violence takes place.[48]

Limited mobility and contact with other people contribute to mental health concerns such as anxiety and depression and further reduce willingness to seek care.[1 44] Lockdown led to decreased freedom and privacy, with attendant physical and psychological stress and fewer sources of social support.[1 9 49] Isolated and unable to seek help,[6 49] women had fewer opportunities to report violence or to escape the situation.[1 4] Although online platforms and mobile applications that enable survivors to chat and seek help have promoted access to support,[50] survivors isolating with family or sharing their devices face constraints to communication by phone,[44] and surveillance of their social media and internet use.[4 47 49] They may also have little time for themselves due to the increase in responsibilities and the small homes common in urban informal settlements. Added to this is some reticence to seek healthcare or alternative shelter for fear of contracting Covid-19.[44] If women do seek care, service provision is itself compromised by closures.[1 9 51]

Burden of unpaid care

The intersection between women's unpaid care work and violence, underpinned by social norms, has recently been highlighted in India.[52] Gender roles pervade experiences of domestic violence,[7 53 54] predominantly by way of family and social expectations. Sexism organises power in families,[55] the Covid-19 pandemic has increased the need for unpaid care and domestic work,[1 6 17 56] and women's perceived failure to meet the increased family obligations has resulted in abuse.[14]

Feminist political economy suggests that these outcomes were predictable: gendered division of labour in private and public domains define how women can earn a living.[57] The expectation that women perform unpaid domestic labour and that their access to paid work and education is undervalued sets the scene for the increased burden and sources of disagreement over the balance between domestic and remunerated work reported in our study. For many of our clients, the level of housework (often falling entirely on wives and daughters) increased, making it more difficult to complete. Some households divided duties such that women were to remain home while men left for supplies,[58] barring these women from accessing public channels for survivor support amidst an already diminished support landscape.[1 4 9 28] Added to this is the disproportionate effect of the pandemic on opportunities for women and girls, with drastic reductions in women's sources of work in agriculture and the informal sector reported in Nepal and Pakistan.[7 53]

In addition to their immediate health and safety, the needs born of these circumstances also cause concern for the long-term impacts on gender equality, particularly in terms of women's and girls' access to education and independence. This is illustrated by the rapidity with which lockdown returned women and girls to extended family contexts and the prioritising of housework above schoolwork and remunerated work.[59] Pressure to marry and depletion of work opportunities threaten women's educational prospects and their ability to sustain themselves independently.[29 53]

Limitations

We do not know whether, at population level, the response to the pandemic increased the incidence or intensity of violence against women and girls. All our respondents had suffered physical, sexual, emotional, or economic violence and had previously reached a point at which they had consulted a counsellor. They were among the minority of survivors who had made the decision to share their concerns outside the home. This may partly explain the pragmatism that characterised their accounts of the situation during lockdown. They had already begun to think through and enact safety plans and coping strategies and violence was not their primary concern in this new and stressful environment.

Many of the factors influencing survivors' experiences, such as the reasons why some families enduring financial hardship would grow closer and some would experience more

For peer review only

tension, are beyond our data. Methodological limitations include the fact that interviews relied on clients' access to phones and sufficient privacy to provide full and accurate details of their situation. Access to both of these is more limited during lockdown and may have affected the quality of answers given the increased presence of household members. Data quality and depth also varied between interviewers, who were collecting information within their counselling sessions.

Recommendations

It was and is difficult for policymakers and civil society organisations to respond to the needs of survivors of violence against women and girls during a pandemic. Our findings highlight the critical role of financial and social support, the need to factor domestic violence into response planning, and the importance of support networks.

Finance and food security are critical

Violence against women intersects with a lack of access to money, food,[60] and (for some) shelter. Any response to the current or future threat to public health must prioritise meeting these needs through economic and livelihood support,[42 51 61] simultaneously reducing the need to leave home to find food, rations, or work.[25 62] The two key sources of disadvantage are poverty and gender. To address these sources, future strategies must include community leaders in the design and implementation of pandemic management strategies and activities.

Support networks are important

Our findings underline the central role of support networks that can identify survivors of violence and help them communicate with services.[17] It may be necessary for service providers to pivot to provide individual and group communication electronically rather than face-to-face. Civil society organisations could expand their remit in two areas. First, preventive counselling to promote healthy relationships and attitudes, beliefs, and behaviours among survivors and perpetrators, and foster problem-solving skills. Second, prevention programs that make communities more aware of their role in identifying and intervening in cases of intimate-partner and domestic violence. It is especially important to take an approach that addresses harmful gendered norms and expectations due to the nature of violence in this context.[52] Current efforts to address domestic violence during the Covid-19 pandemic, including safe-word alert systems, digital or online communications and services, and remote counselling,[50 63] face challenges in our context. For example, a number of respondents were isolated by abusers who used lockdown to control their movement. This prevented them from accessing pharmacies and shops where a safe-word system is typically implemented.

Domestic violence must be factored into response planning

Government and civil society must prioritise the continued provision of support for survivors of violence against women and girls.[17 42] Women need to know that help is available, how to get it, and that their abusers cannot take advantage of social changes to increase the frequency and intensity of violence.[17] This means that services for survivors of violence must be included in national preparedness, response, and recovery plans.[51 56] Responsive funding must be available and be deliverable to the third sector.[51] If programs are to provide more holistic health protection for survivors of domestic violence, they must involve them in planning. Awareness of the issue is paramount and the media should be enlisted (and step forward, as many have done).[51] Civil society organisations who are engaged in community outreach should continue and augment efforts to mobilise communities around intolerance of violence and include training programs on violence arising during natural disasters. Examples include training public health and law enforcement officials to understand and identify domestic violence,[64] community mobilisation motivated in part or whole by prevention of violence, and allocation of safe shelter for survivors at risk.

For peer review only - http://bmjopen.bmj.com/site/about/guidelines.xhtml

CONCLUSION

In beginning to understand the impact of Covid-19 and subsequent lockdown measures on survivor needs and vulnerabilities, our findings underline the importance of financial precarity and access to resources and support. The response to the epidemic is an increase in existing stressors—particularly poverty and gendered household labour—as well as changes in households and patterns of alcohol use. Programs must focus on structural vulnerability, distributing resource-based support, prioritising the continued availability of services for survivors of violence, and expanding access to support networks. Decision-makers must be aware of the gendered and intersectional effects of interventions and must include survivors of domestic violence in the planning and implementation of public health strategies.

ACKNOWLEDGMENTS

We thank the counsellors who spoke with their clients and collected the information on which the paper was developed. We thank counselling coordinators Jyoti Borkar, Vandana Rokde, Vandana Singh, and Shirisha Yeotikar, counselling and legal coordinator Reshma Jagtap, and associate program director Sangeeta Punekar. Archana Bagra provided financial oversight and Vanessa D'Souza and Shanti Pantvaidya organisational leadership.

FUNDING STATEMENT

This work was done by the NIHR Global Health Research Group on a package of care for the mental health of survivors of violence in South Asia, funded by the UK National Institute for Health Research (17/63/47). The funder had no role in study design, data collection and analysis, decision to publish, or preparation of the manuscript.

Contributorship

ND and TD conceived the study. ND and DO acquired funding. TD and MH developed the methodology. ND, TD, DD, and DO oversaw investigation. MH, TD, and DO curated the data. MH did the first analysis and wrote the first draft. ND managed the project with support from TD. All authors critically reviewed drafts of the manuscript and read and commented on the final version. DO accepts full responsibility for the work and the conduct of the study, had access to the data, and controlled the decision to publish.

Competing interests

ND and TD work with SNEHA, the organisation at which the study was done.

Data availability

Data are available in Open Science Framework: Osrin, D. (2021, September 7). Covid-19 interviews. Retrieved from osf.io/9kg67

Ethics statement

The project was conducted within the NIHR Global Health Research Group on *A package of care for the mental health of survivors of violence in South Asia*, with approval for qualitative data collection from the University College London Research Ethics Committee (2744/007) and the Sangath Institutional Review Board (AN_2018_46). Clients gave signed consent for anonymised or pseudonymised use of their data at initial registration. They were assured of confidentiality, particularly that information would not be shared with their family, community, or the media. Counsellors informed clients of their right to access their records, including for use in building legal evidence.

REFERENCES

1. Hall BJ, Tucker JD. Surviving in place: the coronavirus domestic violence syndemic. *Asian J Psychiatry* 2020;53:102179.

For peer review only - http://bmjopen.bmj.com/site/about/guidelines.xhtml

2. Mazza M, Marano G, Lai C, et al. Danger in danger: Interpersonal violence during COVID-19 quarantine. *Psychiatry Res* 2020;289:113046. doi: 10.1016/j.psychres.2020.113046 [published Online First: 2020/11/27]
3. Evans ML, Lindauer M, Farrell ME. A Pandemic within a Pandemic - Intimate Partner Violence during Covid-19. *N Engl J Med* 2020;383(24):2302-04. doi: 10.1056/NEJMp2024046 [published Online First: 2020/09/17]
4. Bradbury-Jones C, Isham L. The pandemic paradox: The consequences of COVID-19 on domestic violence. *J Clin Nurs* 2020;29(13-14):2047-49. doi: 10.1111/jocn.15296 [published Online First: 2020/04/14]
5. UN Women. The shadow pandemic: violence against women during COVID-19 2021 [Available from: https://www.unwomen.org/en/news/in-focus/in-focus-gender-equality-in-covid-19-response/violence-against-women-during-covid-19 accessed 8 Feb 2021.
6. Viero A, Barbara G, Montisci M, et al. Violence against women in the Covid-19 pandemic: A review of the literature and a call for shared strategies to tackle health and social emergencies. *Forensic Sci Int* 2020;319:110650. doi: 10.1016/j.forsciint.2020.110650 [published Online First: 2020/12/20]
7. Sah LK, Sah P, Yadav MK, et al. Risk of gender-based violence and poor mental health among Nepalese women during COVID-19 pandemic: a review. *Nepal Med J* 2020;3(2):39-46.
8. UN Women. Impact of COVID-19 on violence against women and girls and service provision: UN Women rapid assessment and findings. COVID-19 Response. New York NY: United Nations Women, 2020.
9. Sánchez OR, Vale DB, Rodrigues L, et al. Violence against women during the COVID-19 pandemic: An integrative review. *Int J Gynaecol Obstet* 2020;151(2):180-87. doi: 10.1002/ijgo.13365 [published Online First: 2020/09/04]
10. Pfitzner N, Fitz-Gibbon K, True J. Responding to the ‘shadow pandemic’: practitioner views on the nature of and responses to violence against women in Victoria, Australia during the COVID-19 restrictions. Clayton VIC: Monash University, 2020.
11. Hsu LC, Henke A. COVID-19, staying at home, and domestic violence. *Rev Econ Househ* 2020;Nov 20:1-11.
12. Agüero JM. COVID-19 and the rise of intimate partner violence. *World Dev* 2021;137:105217. doi: 10.1016/j.worlddev.2020.105217 [published Online First: 2020/10/06]
13. Walklate S, Godfrey B, Richardson J. Changes and continuities in police responses to domestic abuse in England and Wales during the Covid-19 ‘lockdown’. *Policing and Society* 2021:1-13. doi: 10.1080/10439463.2021.1896514
14. Pinchoff J, Austrian K, Rajshekhar N, et al. Gendered economic, social and health effects of the COVID-19 pandemic and mitigation policies in Kenya: evidence from a prospective cohort survey in Nairobi informal settlements. *BMJ open* 2021;11(3):e042749. doi: 10.1136/bmjopen-2020-042749 [published Online First: 2021/03/05]
15. Sharma P, Khokhar A. Domestic violence and coping strategies among married adults during lockdown due to Coronavirus disease (COVID-19) pandemic in India: a cross-sectional study. *Disaster Med Public Health Prep* 2021:1-29. doi: 10.1017/dmp.2021.59 [published Online First: 2021/03/04]
16. Fraser E. Impact of COVID-19 pandemic on violence against women and girls. VAWG Helpdesk Research Report No 284. London: VAWG Helpdesk, 2020.
17. World Bank Group. Gender dimensions of the COVID-19 pandemic. Policy Notes. Washington DC: World Bank, 2020.
18. Majumdar S, Wood G. UNTF EVAW Briefing Note on the impact of COVID-19 on violence against women through the lens of Civil Society and Women’s Rights Organizations. New York NY: UN Trust Fund to End Violence Against Women, 2020.

For peer review only - http://bmjopen.bmj.com/site/about/guidelines.xhtml

19. Gosangi B, Park H, Thomas R, et al. Exacerbation of Physical Intimate Partner Violence during COVID-19 Pandemic. *Radiology* 2021;298(1):E38-e45. doi: 10.1148/radiol.2020202866 [published Online First: 2020/08/14]
20. Evans DP. COVID-19 and violence: a research call to action. *BMC Womens Health* 2020;20(1):249. doi: 10.1186/s12905-020-01115-1 [published Online First: 2020/11/12]
21. WHO. COVID-19 and violence against women. What the health sector/system can do. Geneva: World Health Organization, 2020.
22. Vora M, Malathesh BC, Das S, et al. COVID-19 and domestic violence against women. *Asian J Psychiatr* 2020;53:102227-27. doi: 10.1016/j.ajp.2020.102227 [published Online First: 2020/06/15]
23. Krishnakumar A, Verma S. Understanding Domestic Violence in India During COVID-19: a Routine Activity Approach. *Asian J Criminol* 2021:1-17. doi: 10.1007/s11417-020-09340-1 [published Online First: 2021/03/17]
24. United Nations Human Settlements Programme (UN-Habitat). The challenge of slums: global report on human settlements 2003. London and Sterling VA: Earthscan Publications Ltd 2003.
25. Golechha M. COVID-19 Containment in Asia's Largest Urban Slum Dharavi-Mumbai, India: Lessons for Policymakers Globally. *J Urban Health* 2020;97(6):796-801. doi: 10.1007/s11524-020-00474-2 [published Online First: 2020/08/21]
26. Slater J, Masih N, Parth MN. In one of the world's largest slums, the fight against the coronavirus has turned into a struggle to survive. *Washington Post* 2020 12 May 2020.
27. Ray D, Subramanian S, Vandewalle L. India's lockdown. CEPR Policy Insight No 102. London: Centre for Economic Policy Research, 2020.
28. Mahapatro M, Prasad MM, Singh SP. Role of Social Support in Women facing Domestic Violence during Lockdown of Covid-19 while Cohabiting with the Abusers: Analysis of Cases Registered with the Family Counseling Centre, Alwar, India. *J Family Issues* 2021;January 2021:0192513X20984496. doi: 10.1177/0192513x20984496
29. Singh S, Bhattacharyya R. A review of domestic violence against women in India during lockdown. *Int J Innovation Creativity Change* 2020;14(6)
30. UN Women. COVID-19 and essential services provision for survivors of violence against women and girls. COVID-19 Response. New York NY: United Nations Women, 2020.
31. Bourgault S, Peterman A, O'Donnell M. Violence against women and children during COVID-19—one year on and 100 papers in. Washington DC: Center for Global Development, 2021.
32. Daruwalla N, Kanougiya S, Gupta A, et al. Prevalence of domestic violence against women in informal settlements in Mumbai, India: a cross-sectional survey. *BMJ open* 2020;10(12):e042444. doi: 10.1136/bmjopen-2020-042444 [published Online First: 2020/12/18]
33. Ritchie J, Spencer L. Qualitative data analysis for applied policy research. In: Bryman A, Burgess RG, eds. Analysing qualitative data. London and New York: Routledge 1994:173-94.
34. Gale NK, Heath G, Cameron E, et al. Using the framework method for the analysis of qualitative data in multi-disciplinary health research. *BMC Med Res Methodol* 2013;13:117. doi: 10.1186/1471-2288-13-117 [published Online First: 2013/09/21]
35. Srivastava A, Thomson SB. Framework Analysis: a qualitative methodology for applied policy research. *Journal of Administration & Governance* 2009;4(2):72-79.
36. Attride-Stirling J. Thematic networks: an analytic tool for qualitative research. *Qualitative Research* 2001;1(3):385-405. doi: 10.1177/146879410100100307
37. Lacey A, Luff D. Qualitative data analysis. Yorkshire & the Humber: National Institute for Health Research 2007.
38. Pope C, Ziebland S, Mays N. Qualitative research in health care: analysing qualitative data. *British Medical Journal* 2000;320:114-16.

For peer review only - http://bmjopen.bmj.com/site/about/guidelines.xhtml

39. Smith J, Firth J. Qualitative data analysis: the framework approach. *Nurse Researcher* 2011;18:52-62.
40. Renzetti CM, Campe M. Feminist praxis and gender violence. In: Naples NA, ed. Companion to feminist studies. Chichester: Wiley-Blackwell 2020:411-26.
41. Nigam S. COVID-19: India's response to domestic violence needs rethinking. *South Asia J* 2020;Available at SSRN: https://ssrn.com/abstract=3598999
42. Hamadani JD, Hasan MI, Baldi AJ, et al. Immediate impact of stay-at-home orders to control COVID-19 transmission on socioeconomic conditions, food insecurity, mental health, and intimate partner violence in Bangladeshi women and their families: an interrupted time series. *The Lancet Global health* 2020;8(11):e1380-e89. doi: 10.1016/s2214-109x(20)30366-1 [published Online First: 2020/08/29]
43. Al-Ali N. Covid-19 and feminism in the Global South: Challenges, initiatives and dilemmas. *European Journal of Women's Studies* 2020;27(4):333-47. doi: 10.1177/1350506820943617
44. Moreira DN, Pinto da Costa M. The impact of the Covid-19 pandemic in the precipitation of intimate partner violence. *Int J Law Psychiatry* 2020;71:101606. doi: 10.1016/j.ijlp.2020.101606 [published Online First: 2020/08/10]
45. Jarnecke AM, Flanagan JC. Staying safe during COVID-19: How a pandemic can escalate risk for intimate partner violence and what can be done to provide individuals with resources and support. *Psychol Trauma* 2020;12(S1):S202-s04. doi: 10.1037/tra0000688 [published Online First: 2020/06/20]
46. Akel M, Berro J, Rahme C, et al. Violence Against Women During COVID-19 Pandemic. *Journal of Interpersonal Violence* 2021:0886260521997953. doi: 10.1177/0886260521997953
47. Ragavan MI, Garcia R, Berger RP, et al. Supporting Intimate Partner Violence Survivors and Their Children During the COVID-19 Pandemic. *Pediatrics* 2020;146(3) doi: 10.1542/peds.2020-1276 [published Online First: 2020/08/21]
48. Felson M, Cohen LE. Human ecology and crime: a routine activity approach. *Human Ecology* 1980;8:389-406.
49. Campbell AM. An increasing risk of family violence during the Covid-19 pandemic: strengthening community collaborations to save lives. *Forensic Sci Int* 2020;Reports 2:100089.
50. Emezue C. Digital or digitally delivered responses to domestic and intimate partner violence during COVID-19. *JMIR Public Health Surveill* 2020;6:e19831.
51. UN Women. Prevention: violence against women and girls & COVID-19. COVID-19 Response. New York NY: United Nations Women, 2020.
52. Oxfam India. On women's backs: India inequality report 2020. New Delhi: Oxfam India, 2020.
53. Malik S, Naeem K. Impact of COVID-19 pandemic on women: health, livelihoods & domestic violence. Islamabad: Sustainable Development Policy Institute, 2020.
54. Agarwal B. Reflections on the Less Visible and Less Measured: Gender and COVID-19 in India. *Gender & Society* 2021;35(2):244-55. doi: 10.1177/08912432211001299
55. George J, Stith SM. An updated feminist view of intimate partner violence. *Fam Process* 2014;53(2):179-93. doi: 10.1111/famp.12073 [published Online First: 2014/04/23]
56. UN Women. From insights to action: gender equality in the wake of COVID-19. COVID-19 Response. New York NY: United Nations Women, 2020.
57. True J. The political economy of violence against women. Oxford: Oxford University Press 2012.
58. Maji S, Bansod S, Singh T. Domestic violence during COVID-19 pandemic: The case for Indian women. *J Community Appl Soc Psychol* 2021 doi: 10.1002/casp.2501 [published Online First: 2021/04/07]
59. Roy AS, Sen N, Bagchi SS. Gender-based violence in India in Covid-19 lockdown. *J Comparative Lit Aesthetics* 2021;44:41-55.

For peer review only - http://bmjopen.bmj.com/site/about/guidelines.xhtml

60. Schokkenbroek JM, Anrijs S, Ponnet K, et al. Locked Down Together: Determinants of Verbal Partner Violence During the COVID-19 Pandemic. *Violence Gender* 2021(5 Apr 2021) doi: 10.1089/vio.2020.0064
61. Béland L-P, Brodeur A, Haddad J, et al. COVID-19, family stress and domestic violence: remote work, isolation and bargaining power. IZA DP No 13332. Bonn: Institute of Labor Economics, 2020.
62. Chakraborty P. Distribution of fresh fruits and vegetables in Dharavi, India: lessons and learnings from the Covid-19 pandemic. Mumbai: Society for Nutrition, Education and Health Action, 2020.
63. Natarajan J. Women's safety during lockdown. *The Hindu* 2020 13 April 2020.
64. Peterman A, Potts A, O'Donnell M, et al. Pandemics and violence against women and children. Working Paper 528. Washington DC: Center for Global Development, 2020.

For peer review only

For peer review only - http://bmjopen.bmj.com/site/about/guidelines.xhtml

SNEHA Program on Prevention of Violence Against Women and Children

Counsellor telephone interviews with registered women clients

March 2020

Topic guide

We are interested in understanding how the Covid-19 situation has affected our clients.

During your phone conversation with the client, please ask her the series of questions below. Record or note what she says. Before you do this, take her consent for the recording (see below).

We are doing this for the NIHR Global Health Research Group on a package of care for the mental health of survivors of violence in South Asia.

We have approval for qualitative data collection from the University College London Research Ethics Committee (2744/007) and the Sangath Institutional Review Board (AN_2018_46).

Please remind the client that when she first consulted SNEHA she agreed that we could use information she provided for research purposes. We will make sure that anything she says is not identifiable and we will never include her name or other details in any information we share. We will use the information collected to improve the programme in times of pandemics like Covid-19 in the future. You can assure the client that her information will be kept anonymous and confidential and only used by the program.

Tell the client that she does not have to answer any question if she doesn't want to, and that whether or not she does will have no bearing on the way SNEHA supports her.

Ask her if she agrees to either:

Her answers to the questions being recorded on the phone.

Or:

You taking notes of the answers to the questions.

The questions

You may like to ask these questions early in the conversation as they are more general and less sensitive than the issues for which she has consulted and which you will be discussing during the call.

You can ask the questions later if the client wants to discuss a specific issue about her case. If she would prefer it, you can ask her for an exclusive appointment later to ask the interview questions.

You don't have to ask them in order or exactly as they are written: listen to what the client says and then probe until you understand.

How are you doing in the present situation?

Are you facing any kind of problems?

Has the Covid-19 crisis made your situation different from earlier times in your day-to-day life?

How is it with everyone staying at home?

Have your responsibilities changed compared to before the situation?

How are you managing things like food and money?

How is your family reacting to the situation?

Do you think the situation has affected you being able to talk to someone about your issues?

Has the crisis brought your family closer or has it created more problems?

Do you think counselling could be done better if you went to the counselling centre instead of talking like this on the phone?

Note down any particular observations or difficulties you have in the conversation.

For peer review only

COREQ (COnsolidated criteria for REporting Qualitative research) Checklist

A checklist of items that should be included in reports of qualitative research. You must report the page number in your manuscript where you consider each of the items listed in this checklist. If you have not included this information, either revise your manuscript accordingly before submitting or note N/A.

Topic	Item No.	Guide Questions/Description	Reported on Page No.
**Domain 1: Research team and reflexivity**			
Personal characteristics			
Interviewer/facilitator	1	Which author/s conducted the interview or focus group?	5
Credentials	2	What were the researcher's credentials? E.g. PhD, MD	5
Occupation	3	What was their occupation at the time of the study?	5
Gender	4	Was the researcher male or female?	5
Experience and training	5	What experience or training did the researcher have?	5
Relationship with participants			
Relationship established	6	Was a relationship established prior to study commencement?	5
Participant knowledge of the interviewer	7	What did the participants know about the researcher? e.g. personal goals, reasons for doing the research	5
Interviewer characteristics	8	What characteristics were reported about the inter viewer/facilitator? e.g. Bias, assumptions, reasons and interests in the research topic	5
**Domain 2: Study design**			
Theoretical framework			
Methodological orientation and Theory	9	What methodological orientation was stated to underpin the study? e.g. grounded theory, discourse analysis, ethnography, phenomenology, content analysis	5
Participant selection			
Sampling	10	How were participants selected? e.g. purposive, convenience, consecutive, snowball	5
Method of approach	11	How were participants approached? e.g. face-to-face, telephone, mail, email	5
Sample size	12	How many participants were in the study?	6
Non-participation	13	How many people refused to participate or dropped out? Reasons?	6
Setting			
Setting of data collection	14	Where was the data collected? e.g. home, clinic, workplace	5
Presence of non-participants	15	Was anyone else present besides the participants and researchers?	5
Description of sample	16	What are the important characteristics of the sample? e.g. demographic data, date	6
Data collection			
Interview guide	17	Were questions, prompts, guides provided by the authors? Was it pilot tested?	5
Repeat interviews	18	Were repeat inter views carried out? If yes, how many?	0
Audio/visual recording	19	Did the research use audio or visual recording to collect the data?	0
Field notes	20	Were field notes made during and/or after the inter view or focus group?	5
Duration	21	What was the duration of the inter views or focus group?	5
Data saturation	22	Was data saturation discussed?	0
Transcripts returned	23	Were transcripts returned to participants for comment and/or	0

For peer review only

Topic	Item No.	Guide Questions/Description	Reported on Page No.
		correction?	
**Domain 3: analysis and findings**			
Data analysis			
Number of data coders	24	How many data coders coded the data?	5
Description of the coding tree	25	Did authors provide a description of the coding tree?	5
Derivation of themes	26	Were themes identified in advance or derived from the data?	5
Software	27	What software, if applicable, was used to manage the data?	5
Participant checking	28	Did participants provide feedback on the findings?	0
Reporting			
Quotations presented	29	Were participant quotations presented to illustrate the themes/findings? Was each quotation identified? e.g. participant number	6-8
Data and findings consistent	30	Was there consistency between the data presented and the findings?	6-8
Clarity of major themes	31	Were major themes clearly presented in the findings?	6-8
Clarity of minor themes	32	Is there a description of diverse cases or discussion of minor themes?	9

Developed from: Tong A, Sainsbury P, Craig J. Consolidated criteria for reporting qualitative research (COREQ): a 32-item checklist for interviews and focus groups. *International Journal for Quality in Health Care*. 2007. Volume 19, Number 6: pp. 349 – 357

Once you have completed this checklist, please save a copy and upload it as part of your submission. DO NOT include this checklist as part of the main manuscript document. It must be uploaded as a separate file.

For peer review only
